# Complete CD16A Deficiency and Defective NK Cell Function in a Man Living with HIV

**DOI:** 10.1007/s10875-025-01886-y

**Published:** 2025-05-24

**Authors:** Weiying Zhang, Alan F. Scott, David W. Mohr, Roxann Ingersoll, Peter E. Shoucair, Jay H. Bream, Tricia L. Nilles, Hao Zhang, Yue Chen, Robbie B. Mailliard, Joseph B. Margolick

**Affiliations:** 1 Department of Molecular Microbiology and Immunology, Johns Hopkins Bloomberg School of Public Health, 615 N Wolfe St., Baltimore, MD 21205 USA; 2 Department of Genetic Medicine, Johns Hopkins School of Medicine, Baltimore, MD USA; 3 Department of Medicine, University of Pittsburgh School of Medicine, Pittsburgh, PA USA; 4 Graduate Program in Immunology, Johns Hopkins School of Medicine, Baltimore, MD USA

**Keywords:** CD16A deficiency, FCGR3A deletion, NK cell function, Monocyte function, Dendritic cell function

## Abstract

A man living with HIV was found to lack expression of CD16A on his natural killer (NK) cells and monocytes. Genetic analysis revealed compound heterozygous deletion of *FCGR3A*, the gene encoding CD16A. The case’s NK cells showed: (a) no antibody-dependent cell-mediated cytotoxicity and very low spontaneous cytotoxicity; (b) an immature phenotype marked by high expression of CD94, CD2, NKG2A, and NKG2D, and low expression of KIR2DL2 and CD57; (c) no expression of KIR3DL1 and very low expression of FcRγ; and (d) normal cytokine production. The case’s monocytes and DCs were similar phenotypically and functionally to those from the donors matched for HIV status, age, and percentage of NK cells in the peripheral blood. In contrast to previously reported people with CD16A deficiency, this man did not have a history of severe infections with herpes viruses, suggesting that other immune cells and/or immunoregulatory function of NK cells may compensate for deficiency of cytolytic NK cells.

**Supplementary Information:**

The online version contains supplementary material available at 10.1007/s10875-025-01886-y.

## Introduction

The CD16 protein (Fcγ-Receptor III (FcγRIII)) binds with low affinity to the Fc region of the IgG antibody molecule [1, 2]. Its two isoforms, CD16A and CD16B, are encoded by paralogous genes, *FCGR3A* and *FCGR3B*, respectively. CD16A is expressed on monocytes, dendritic cells (DCs), macrophages [1, 3] and NK cells, its activation is important for antibody-dependent cell-mediated cytotoxicity (ADCC) [2, 4], spontaneous cytotoxicity [5, 6], and secretion of interferon-γ (IFN-γ) and tumor necrosis factor-α (TNF-α) [7]. For DCs and mononuclear phagocytes, CD16A activation has been associated with opsonophagocytosis, ADCC, production of inflammatory cytokines, and enhanced antigen presentation [3]. CD16B, expressed on neutrophils and subsets of basophils [1, 8], has been considered a decoy receptor that competes for immune complexes [2] and mediates trogocytosis of opsonized target cells [9].

*FCGR3A* and *FCGR3B* are located in the FCGR2/3 locus at chromosome 1q23.3, where high homology between two tandem paralogous blocks renders them susceptible to deletion or duplication by non-allelic homologous recombination [10, 11], leading to copy number variations [12, 13]. Having only 1 copy of *FCGR3A* has been associated with lower ADCC activity of NK cells [12] and with clinical conditions, e.g., sarcoidosis [14] and systemic lupus erythematosus (SLE) [15, 16]. Having 0 or 1 copy of *FCGR3B* has been associated with SLE and rheumatoid arthritis (RA) [1, 15, 17]. A recently reported person with deletion of FCRG3A in one chromosome and fusion of *FCGR3A* with *FCGR3B* in the other lacked CD16A on NK cells and NK cell-mediated ADCC, had persistent Epstein Barr virus (EBV) infection and renal failure, and had abnormally high percentages of NK cells with an early-differentiated phenotype (CD56^dim^NKG2A^+^NKG2D^+^CD94^+^CD57^−^KIR^−^) associated with acute EBV infection [17], and low percentages of memory-like or adaptive (CD56^dim^CD57^+^NKG2C^+^ and CD56^dim^CD57^+^FcRγ^low/−^) NK cells [18]. This reported case suggests that absence of CD16A affects development of NK cells.

Here we report a man with no expression of CD16A on circulating leukocytes due to a compound heterozygous deletion of *FCGR3A*. He has no NK cell-mediated ADCC and defective NK-mediated cytotoxicity but has remained healthy with virologically suppressed HIV infection.

## Methods

### Study Subject

The case is an African-American man enrolled in the Multicenter AIDS Cohort (MACS), a study of HIV infection in men who have sex with men ongoing in 4 US sites since 1984 [19]. MACS participants have semiannual study visits at which medical and behavioral history are recorded, physical exam and laboratory testing are performed, and plasma, serum, and viable peripheral blood mononuclear cells (PBMC) are frozen [19]. In another study, cryopreserved PBMC from this man surprisingly did not express CD16 by flow cytometry. Therefore, further studies were undertaken.

At enrollment in the MACS in 2003, the case was 42 years old, was receiving combination antiretroviral therapy (cART), and was virologically suppressed (plasma HIV RNA < 50 copies/ml by Roche ultrasensitive assay (Roche Diagnostics, Nutley, NJ)), with normal white blood count, hemoglobin A1c concentration, and serum concentration of alkaline phosphatase (Table 1). Later, when the MACS performed more extensive testing, he had slightly elevated creatinine, alkaline phosphatase, and gamma-glutamyl transferase in serum and total protein in urine but did not show any signs of immunodeficiency or persistent infections. He had cleared infection with hepatitis B virus and no infection with hepatitis C virus. His HIV-related history (Table 2) showed undetectable plasma HIV RNA at most study visits, with CD4 T cell counts generally > 500/µL when he was virologically suppressed. Intermittent non-suppression of HIV replication was possibly due to reduced adherence to antiretroviral therapy (Table 2). At both study entry and at follow-up study visits he reported no hospitalizations or other illnesses.

**Table 1:** Laboratory data of the case

	Time of study enrollment	A study visit 10 years later	Normal range
Age (yr)	42	52	
Hemoglobin A1C (% of total Hgb)	4.7	5.1	< 5.7
Absolute neutrophil count (cells/uL)		3600	1500–7800
White blood count (1000 cells/uL)	7.6	7.2	3.8–10.8
Alkaline phosphatase (U/L)	68	**128**	40–115
Lactate dehydrogenase (U/L)		183	120–250
Gamma-glutamyl transferase (U/L)		**158**	3–85
Creatinine (mg/dL)		1.19	0.7–1.33
Urea nitrogen (mg/dL)		20	7–25
Creatinine, urine (mg/dL)		**375** ^a^	20–370
Total protein, urine (mg/dL)		**51**	5–25
Protein/Creatinine ratio, urine (mg/g creatinine)		**136**	22–128
Estimated Glomerular Filtration Rate (mL/min/1.73m^2^)		86	≥60
Complement Total (CH50) (U/mL)		58	31–60
Complement component C3C (mg/dL)		121	82–185
Complement component C4C (mg/dL)		33	15–53
Immunoglobulin A (mg/dL)		312	70–320
Immunoglobulin M (mg/dL)		70	50–300
Immunoglobulin G (mg/dL)		1486	600–1540
Immunoglobulin G subclass 1 (mg/dL)		**986**	382–929
Immunoglobulin G subclass 2 (mg/dL)		314	241–700
Immunoglobulin G subclass 3 (mg/dL)		78	22–178
Immunoglobulin G subclass 4 (mg/dL)		30	4–86

^a^ Values in **bold** are out of the normal ranges

**Table 2:** HIV-related laboratory data of the case

Age	%CD4	CD4 cell count (cells/mm^3^)	CD8 cell count (cells/mm^3^)	CD4/CD8 ratio	Plasma HIV RNA (copies/mL)	Antiretroviral treatment	Self-reported treatment adherence^c^
42^a^	37	1095	1214	0.9	< 50	Potent ART^b^	95–99%
43	39	948	948	1	< 50	Potent ART	95–99%
43	37	730	809	0.9	< 50	Potent ART	95–99%
44	39	608	593	1.03	< 50	Potent ART	95–99%
44	22	319	682	0.47	21,300	Potent ART	100%
45	30	496	760	0.65	< 50	Potent ART	100%
45	35	558	637	0.88	< 50	Potent ART	95–99%
46	40	749	711	1.05	< 50	Potent ART	95–99%
46	42	511	462	1.11	< 50	*Potent ART*	100%
47	40	896	874	1.03	< 50	Potent ART	100%
47	41	722	616	1.17	< 50	Potent ART	100%
48	38	635	669	0.95	< 50	Potent ART	100%
48	41	852	748	1.14	< 50	Potent ART	95–99%
49	35	551	646	0.85	< 50	Potent ART	100%
49	38	693	766	0.9	< 50	Potent ART	95–99%
50	30	445	668	0.67	30,300	Potent ART	100%
50	22	396	881	0.45	28,400	Potent ART	100%
51	26	508	879	0.58	27,600	Potent ART	95–99%
51	27	611	1086	0.56	311	Potent ART	75–94%
52^a^	40	1119	1037	1.05	< 50	Potent ART	100%
53^a^	40	1056	1030	1	< 50	Potent ART	95–99%
53	28	782	1403	0.54	44,457	No Therapy	< 75%
54	35	975	1165	0.81	< 50	Potent ART	95–99%
54	38	1256	1454	0.86	< 50	Potent ART	95–99%
55	41	1212	1126	1.05	< 50	Potent ART	100%
56	39	1318	1285	1	< 50	Potent ART	100%
57	36	1029	1307	0.77	< 50	Potent ART	100%
58^a^	43	1413	1188	1.19	< 50	Potent ART	100%

^a^ Indicates the study visits from which specimens were taken for the analysis of CD16A expression and the phenotype and function of NK cells

^b^ Potent antiretroviral therapy contained two nucleoside reverse transcriptase inhibitors (NRTIs) and a non-nucleoside reverse transcriptase inhibitor (NNRTI) at all visits except the italicized visit, when it was switched to three NRTIs. ^c^ Self-reported adherence in the last 4 days was recorded by adherence questionnaire administered at each visit [20]

After identification of his CD16 deficiency, when he was 61 years old, a more detailed medical and family history was obtained, and serum concentrations of total complement, complement components C3C and C4C, IgG and its subclasses, IgM, and IgA were measured in stored serum at Quest Diagnostics (Frederick, MD). He did not recall having had any unusual, serious, or persistent infections, had no history of recurrent warts or herpetic lesions, had never been told of any problems with his immune system, and had no family history of infections or early death, including both parents and a half-sister who was alive and healthy. Serum concentrations of complement and immunoglobulins were normal, except that concentration of IgG1 was slightly higher than the normal range (Table 2).

### Flow Cytometric Analysis of CD16A Expression

CD16A expression was analyzed in PBMC from (a) the case, (b) a healthy HIV-uninfected donor described previously [21], and (c) a MACS participant who is living with HIV and had similar age and percentage of NK cells among peripheral lymphocytes as the case. NK cells (CD45^+^CD3^−^CD56^+^/CD16^+^) and monocytes (CD45^+^CD3^−^HLA-DR^+^CD14^+/−^) [22] in PBMC were identified with monoclonal antibodies (mAbs, from BD Biosciences (San Jose, CA) unless otherwise noted): anti-CD14-brilliant violet (BV) 605, anti-HLA-DR-BV650, anti-CD3-fluorescein isothiocyanate (FITC), anti-CD56/CD16-phycoerythrin (PE), anti-CD45-peridinin-chlorophyll-protein (PerCP), and Near-IR LIVE/DEAD™ Fixable dye (Invitrogen, Carlsbad, CA). Surface expression of CD16 was assessed with two mAbs: anti-CD16-BV421 (clone: B73.1) and anti-CD16-BV510 (clone: 3G8), which identify different epitopes on CD16 [5, 23]. To assess intracellular expression of CD16A, cells stained with all mAbs above except anti-CD16A, fixed and permeabilized with Cytofix/Cytoperm™ (BD Biosciences), and stained with anti-CD16A BV421 and anti-CD16A-BV510. Data were acquired on a LSRII cytometer (BD Biosciences) and analyzed using FlowJo 10.7.1 (FlowJo, Ashland, OR). Gates were set using fluorescence-minus-one (FMO) controls [24], as shown in Fig. S1. PBMC from the case were initially tested in three experiments, using cryopreserved PBMC from 2 study visits 4.5 years apart. Later, we also analyzed CD16A expression on cryopreserved PBMC obtained from the case at his earliest available MACS study visit.

The following mAbs (all from BD Biosciences) were used to assess CD16A expression in NK cells and monocytes: anti-CD3-brilliant ultra violet (BUV)805, anti-CD56-BUV615, anti-CD16 -BV421 (clone: 3G8), anti-CD19-FITC, anti-HLA-DR-PE-Cy7, anti-CD14-Allophycocyanin (APC), anti-CD16-R718 (clone: B73.1), and Yellow LIVE/DEAD™ Fixable dye (Invitrogen). Data were acquired on a FACSymphony™ A5 cytometer (BD Biosciences) and analyzed using FlowJo 10.10.0 (FlowJo).

### Genetic Analyses of *FCGR3A*

Although locus-specific PCR and Sanger sequencing suggested a mutation in the *FCGR3A* and *FCGR3B* loci, because of the high homology between these two genes we chose to use 10X Genomics Chromium™ (10X Genomics, Pleasanton, CA) linked-read sequencing to obtain phased parental reads that would span both loci [25, 26].

High molecular weight genomic DNA (50 kilobase (kb)– 1 + megabase (Mb)) was isolated from thawed PBMC of the case using the Nanobind CBB Big DNA Kit (Circulomics, Baltimore, MD) per manufacturer’s instructions, quantified using a Qubit fluorimeter, and diluted to 1.25 ng/ul in Tris-EDTA (TE) buffer. The DNA was processed using the Chromium platform (10X Genomics) and a sequencing library prepared following the manufacturer’s protocol [27]. The library was quantified both by qPCR and by Bioanalyzer (Agilent, Santa Clara, CA) electrophoresis and sequenced on a NovaSeq 6000 (Illumina, San Diego, CA) to generate 855.8 million reads with a mean read length of 139 bp (bp) after trimming and a mean read depth of 36.7X. Longranger^™^ v.2.2.2 (10X Genomics) was used to phase reads by their shared index. The average molecule length was 67.5 kb, and the N50 phase block size was 14.26 Mb.

The case’s haplotypes were compared to the NA12878 reference genome [28] available from 10X Genomics. Separate deletion events were predicted on each parental chromosome (Fig. 2a) with a retained *FCGR3A*-*3B* fusion allele. We designed PCR primers flanking the retained allele to narrow the cross-over position and performed Sanger sequencing on the products (Fig. 2b). All of the PCR, 10x Genomics, and both types of sequencing were carried out at the JHU *Genetic Resources Core Facility*,* RRID: SCR_018669.*

### Immunophenotyping of NK Cells

Cryopreserved PBMC from the case and 5 other MACS participants matched with the case by HIV status, study visit, age (± 5 years), and percentage of total NK cells (identified as CD45^+^CD3^−^CD16^+^/CD56^+^) among lymphocytes were thawed and stained with the antibodies listed in supplementary Table 1. In experiments 1 and 2, all antibodies were combined in a single panel; however, this panel did not resolve NKG2C well, so in experiment 3 antibodies were divided into 2 panels and anti-NKG2C-PE was used. Each experiment included PBMC from the case (from the same study visit in experiments 1 and 2 and a study visit 4.5 years later in experiment 3) and from two or three matched donors. After surface staining, cells were fixed and permeabilized with eBioscience™ Foxp3 / Transcription Factor Staining Buffer Set (ThermoFisher Scientific, Waltham, MA) per manufacturer’s instructions and stained intracellularly with anti-FcRγ-FITC, anti-TCF-1-PE, and anti-(TBX-21)-PE-Cy7. Data were acquired on a FACSymphony™ A3 cytometer (BD Biosciences) and analyzed using FlowJo 10.7.1 (FlowJo). Gates were set using fluorescence-minus-one controls [24]. Total NK cells were identified as live/dead^−^CD3^−^CD4^−^CD8^−^CD14^−^CD19^−^TBX-21^+^ cells [29]. All NK markers were examined on total NK cells and on CD56^bright^, CD56^dim^, and CD56^neg^ subsets of NK cells to account for differential expression of these markers with differential CD56 expression [30]. In experiment 3 Uniform Manifold Approximation and Projection (UMAP) was used for dimensionality reduction [27], and Flowsom to identify NK cell subsets based on marker expression [31].

### Measurement of NK Cell Function

#### Spontaneous Cytotoxicity and ADCC

Spontaneous cytotoxicity and ADCC of NK cells were assessed in three experiments, each using PBMC from the case (from one study visit) and a matched control. Target cells were K562 cells (American Type Culture Collection (ATCC)-CCL-243, Manassas, VA) for spontaneous cytotoxicity and Raji cells (ATCC-CCL-86) for ADCC. Both cytotoxicities were assessed as described [32–34], except that target cells were labelled with CellTrace™ Violet (Invitrogen) instead of Cell Tracker Orange. Briefly, target cells were labeled with CellTrace™ Violet as described [21]. Labeled Raji cells were incubated with either 10 µg/mL rituximab (generously donated by Genentech, South San Francisco, CA), or human IgG1 (Sigma Aldrich, St. Louis, MO) as a negative control, for one hour at 37 °C. PBMC from the case and matched controls were then mixed in triplicate with target cells at effector: target (E: T) ratios of 20:1, 10:1, 5:1, 2.5:1, and 0:1. After incubation for 4 h at 37 °C, cells were labeled with 1 µg/mL 7-aminoactinomycin D (7-AAD, ThermoFisher Scientific) for 20 min to allow discrimination of dead cells, then analyzed on a FACSCanto II cytometer (BD Biosciences) with dead target cells identified as 7-AAD^+^ CellTrace™Violet^+^. Percent cytotoxicity was then calculated as follows:

% spontaneous cytotoxicity= (% dead K562 cells with PBMC added $\:-\:$% dead K562 cells without PBMC added)/(100 $\:-\:$% dead K562 cells without PBMC added)

%ADCC= (% dead Raji cells with PBMC added to rituximab-coated targets $\:-$% dead Raji cells with PBMC added to IgG1-coated targets)/(100 $\:-\:$% dead Raji cells with PBMC added to IgG1-coated targets)

#### Cytokine Production

NK cells were purified from cryopreserved PBMC by magnetic bead negative selection using a Human NK Cell Isolation Kit (Miltenyi Biotec, Gaithersburg, MD). The phenotypic and functional effects of cytokine treatment of NK cells were assessed as previously described [35, 36]. Briefly, NK cells were seeded in 96-well round-bottom plates at 1 × 10^5^ cells per well, and cultured for 48 h in serum-free AIM V medium (ThermoFisher Scientific) alone, or with addition of interleukin (IL)-18 (1 µg/ml; MBL International, Woburn, MA), or IL-18 in combination with IL-2 (1000 IU/ml; Proleukin; Prometheus Laboratories, San Diego, CA) or with IL-15 (200; R&D Systems, Minneapolis, MN). Cells were stained with mAbs to IFN-γ-AF700 (clone B27, BD Biosciences), MIP-1β-APC (clone D21-1351, BD Biosciences), CD3-APC-H7 (clone SK7, BD Biosciences), CD56-PC7(clone N901, Beckman Coulter, Indianapolis, Indiana) and with Aqua LIVE/DEAD™ Fixable dye (Invitrogen), and then production of IFN-γ and MIP-1β by NK cells was assessed by flow cytometric intracellular cytokine staining as described above for intracellular staining of CD16A. Secretions of IFN-γ and TNF-α into the culture supernatant were assessed by multiplex electrochemiluminescence immunoassay (Meso Scale Diagnostics, Rockville, MD).

### Immunophenotyping of Monocytes and DC

Cryopreserved PBMC from the case and 3 matched controls were thawed and stained with the antibodies listed in supplementary Table 2. Sequential staining of CX3CR1, CCR3, CXCR2, CD14 and CCR2 at 37 °C was performed as described [37] to ensure optimal staining of chemokine receptors on monocytes. Data were acquired on a FACSymphony™ A5 cytometer (BD Biosciences) and analyzed using FlowJo 10.10.0 (FlowJo). Gates were set using FMO controls [24]. Because of the lack of CD16A expression on monocytes of the case, an alternative strategy to identify classical, intermediate, and nonclassical monocytes based on expression of CD14 and CD89 was developed and validated (see details in Supplementary Material).

### Functional Analysis of Monocytes

Monocytes were isolated from cryopreserved PBMC by CD14 positive magnetic beads (Miltenyi Biotec) according to manufacturer’s specifications, and were cultured in Iscove’s Modified Dulbecco’s Media (IMDM; Gibco, ThermoFisher Scientific) containing 10% fetal bovine serum and 0.5% gentamicin (Gibco) in a 24-well flat bottom plate at 5 × 10^5^ cells/well. Following a 24-hour culture in either media alone; IFN-γ (1000 U/mL; R&D Systems) and TLR4 agonist LPS (1ng/ml; Sigma); or IFN-γ (1000 U/mL; R&D Systems) and TLR7 agonist CL307 (750ng/ml; InvivoGen, San Diego, CA), secretions of IL-10, TNF-α, IL-6, and IL-1β into the culture supernatants were measured by multiplex electrochemiluminescence immunoassay (Meso Scale Diagnostics).

### Generation and Functional Analysis of Monocyte-Derived DCs

Monocyte-derived DCs were generated as previously described [38]. Briefly, immature DC were generated by culturing monocytes isolated as described above for 5 days in Iscove’s Modified Dulbecco’s Media (IMDM; Gibco) containing 10% fetal bovine serum and 0.5% gentamicin (Gibco) in the presence of granular-macrophage colony-stimulating factor (GM-CSF) (1000 IU/mL; Sanofi-aventis, Bridgewater, NJ) and IL-4 (1000 IU/mL; R&D Systems). High IL-12p70-producing mature DC were generated by an additional 48 h of culture in the presence of a cocktail of maturation factors containing IFN-α (1000 U/mL; Schering Corporation, Kenilworth, NJ), IFN-γ (1000 U/mL; R&D Systems), IL-1β (10 ng/mL; R&D Systems), TNF-α (25 ng/mL; R&D Systems), and poly-IC (20 ng/mL; Sigma).

Phenotypes of immature and mature monocyte-derived DCs were assessed by staining the cells with anti-CD83-PE (clone HB15e, Beckman Coulter), anti-CD86-PE (clone FUN-1, BD Biosciences), or anti-Siglec-1-PE (clone 7-239, BD Biosciences), followed by data acquisition on a LSRFortessa cytometer (BD Biosciences) and analysis using FlowJo 10.10.0 (FlowJo). Mature DCs were exposed to CD40L-transfected J558 cells (J558-CD40L; a gift from Dr. P. Lane, Birmingham, UK) to induce their production of IL-12p70 as previously described [39]. Briefly, the DC were plated (2.5 × 10^4^ cells/well) in a 96-well flat-bottom plate and stimulated with J558-CD40L (5 × 10^4^ cells/well) for 24 h. Culture supernatants were collected and tested for concentrations of IL-12p70, IL-10, IL-6, IL-8, and TNF-α by multiplex electrochemiluminescence immunoassay (Meso Scale Diagnostics).

### Testing for Antibodies to Herpesviruses

Antibodies to EBV, CMV, herpes simplex virus (HSV)-1/2, varicella-zoster virus (VSV), human herpesvirus (HHV)-6, HHV-7, and HHV-8 were assayed in stored serum using enzyme-linked immunosorbent assays (ELISA) at the National Cancer Institute (Frederick, MD) for antibodies to EBV, CMV, HHV-6 and HHV-8 [40] and at Quest Diagnostics for antibodies to HSV-1/2, VSV, and HHV-7.

### Nucleic Acid Extraction and Quantification of Herpesviruses

Nucleic acid was isolated using a NucliSENS EasyMAG automated extraction system (BioMérieux, Boston, MA) from 1 ml stored plasma spiked with a fixed amount of DNA internal control virus (a complete DNA genome of the phocine (seal) herpes virus type 1, PhHV) [41], and subjected to quantitative real-time PCR targeting EBV, CMV, HHV-6, HHV-8, and PhHV as described [40, 42].

### Statistical Analyses

Differences in spontaneous cytotoxicity and ADCC between the case and the control MACS participants were compared using multilevel mixed effect models, accounting for intra-person and intra-experiment correlations. The non-parametric Mann–Whitney *U* Test was used to compare expression of markers for NK cells, monocyte and DCs between the case and control donors. Analyses were performed using Stata version 15.0 (StataCorp, College Station, TX). A p value of < 0.05 was considered statistically significant.

## Results

### Percentage of NK Cells in the Case and Controls, and Lack of CD16A Expression in PBMC of the Case

The case’s percentage of NK cells among lymphocytes remained consistent across 5 study visits spanning 5 years (median (interquartile range): 4 (1) %, Supplementary Table 3).

**Fig. 1:**
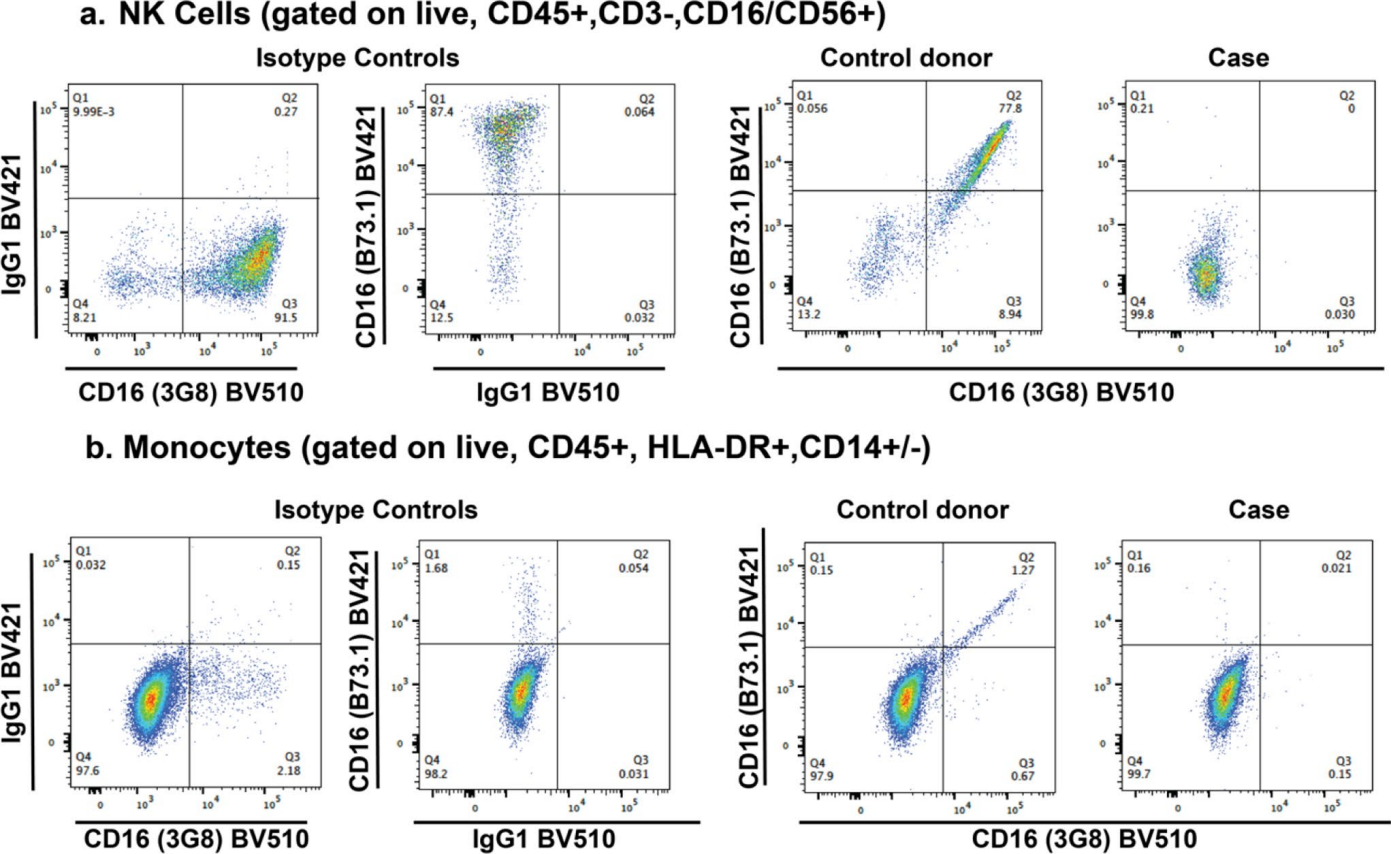
Lack of expression of CD16A on NK cells and monocytes of the case. The flow cytometric plots show the expression of CD16A on (**a**) NK cells and (**b**) monocytes of a healthy HIV-uninfected donor (control donor) and the case using two monoclonal antibodies (mAbs), B73.1 and 3G8, that recognize distinct epitopes of CD16A. NK cells and monocytes were identified as shown in Fig. S1. Gates for identifying CD16A expression were set based on staining of isotype controls (two leftmost columns). CD16A was detected by both mAbs on the cells of the control donor (third column from the left), but not the case (rightmost column) for both NK cells (**a**) and monocytes (**b**)

Both 3G8 and B73.1 anti-CD16A mAbs, which detect different epitopes on CD16A, stained NK cells and monocytes from a control MACS participant as expected (Fig. 1, middle right panels), but not NK cells and monocytes of the case (Fig. 1, lower panels). The same was true for intracellular staining for CD16A in these cells (Fig. S2) and for cells from the earliest sample of PBMC available from the case (Fig. S3).

### Genomic Analysis of *FCGR3A* in the Case

**Fig. 2:**
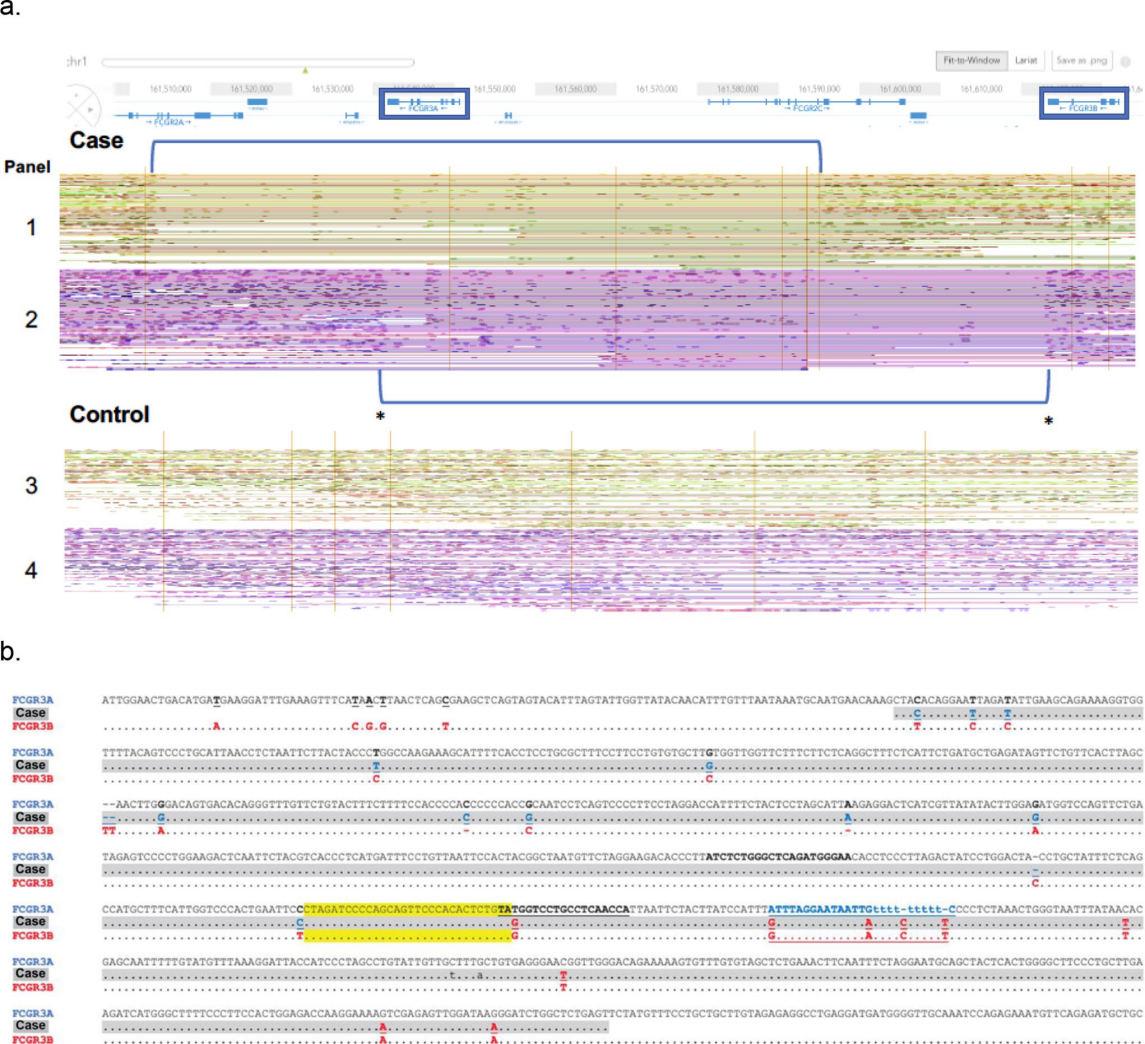
Sequencing results of the *FCGR3A* and *FCGR3B* loci from the case and the human reference sequence NA12878 [28]. **a**. Linked reads aligned around the low/medium-affinity FcγR locus on chromosome 1 with LongRanger™ (10X genomics) and displayed in Loupe™ (10X genomics). The yellow and purple lines represent the phased alignments of the two alleles of the locus in the case (upper pair of yellow and purple lines, panels 1 and 2) and the control (lower pair of yellow and purple lines, representing data from DNA from the cell line NA12878, panels 3 and 4) [28]. Gene regions are indicated above the data for the case, with *FCGR3A* and *FCGR3B* denoted by blue boxes. For the case, both alleles show regions with few, if any, aligned reads indicating deletions (blue brackets). In the yellow allele (panel 1) *FCGR3A* is completely deleted. In the purple allele (panel 2), most of *FCGR3A* and a small part of *FCGR3B* are deleted, as is the intervening DNA between the two genes. These regions, marked with asterisks for the purple allele, indicated a fusion near the 3’ end of each gene. The direction of expression is from right to left. **b**. Sanger sequence of PCR products around the expected breakpoint from the purple allele shown in a), in comparison to the human reference sequences for the *FCGR3A* and *FCGR3B* loci. Nucleotides in *FCGR3B* that differ from those in *FCGR3A* are shown in red. The data narrow the cross-over event to the 30 bp sequence highlighted in yellow, because nucleotides before this region are in agreement with the expected *FCGR3A* sequence (shown in blue letters) while those after this sequence agree with the sequence of *FCGR3B* (shown in red letters). The regions flanking the cross-over region shown in bold represent the location of the primers used for Sanger sequencing. The cross-over falls in the 3’ UTR of *FCGR3B* and replaces it with the sequence from the 3’ UTR of *FCGR3A*

Linked-read sequencing showed that *FCGR3A* was deleted in both parental chromosomes of the case (Fig. 2a, panels 1 and 2) relative to the human gold-standard genome NA12878 (Fig. 2a, panels 3 and 4). However, the deleted regions differed; on one chromosome (Fig. 2a, panel 1) *FCGR3A* was completely missing, while on the other chromosome (Fig. 2a, panel 2) sequencing predicted a fusion of the 3’ end of *FCGR3A* and the 3’ end of *FCGR3B* (asterisks in Fig. 2a, panel 2). This was confirmed by Sanger sequencing a PCR product designed to straddle the junction of the *FCGR3A* and *FCGR3B* genes. Figure 2b shows the expected sequences of *FCGR3A* and *FCGR3B* with the case sequence (greyed) in the line between them. The bases in bold font are the primers used for sequencing the case’s PCR product and the blue or red bases are what are expected for either of the two genes. The case’s sequence shows bases consistent with *FCGR3A* at the 5’ end and with *FCGR3B* at the 3’ end. The 30 bp region highlighted in yellow denotes the region in which the recombination event occurred.

### NK Cell Phenotypes of the Case and Matched Donors

**Fig. 3:**
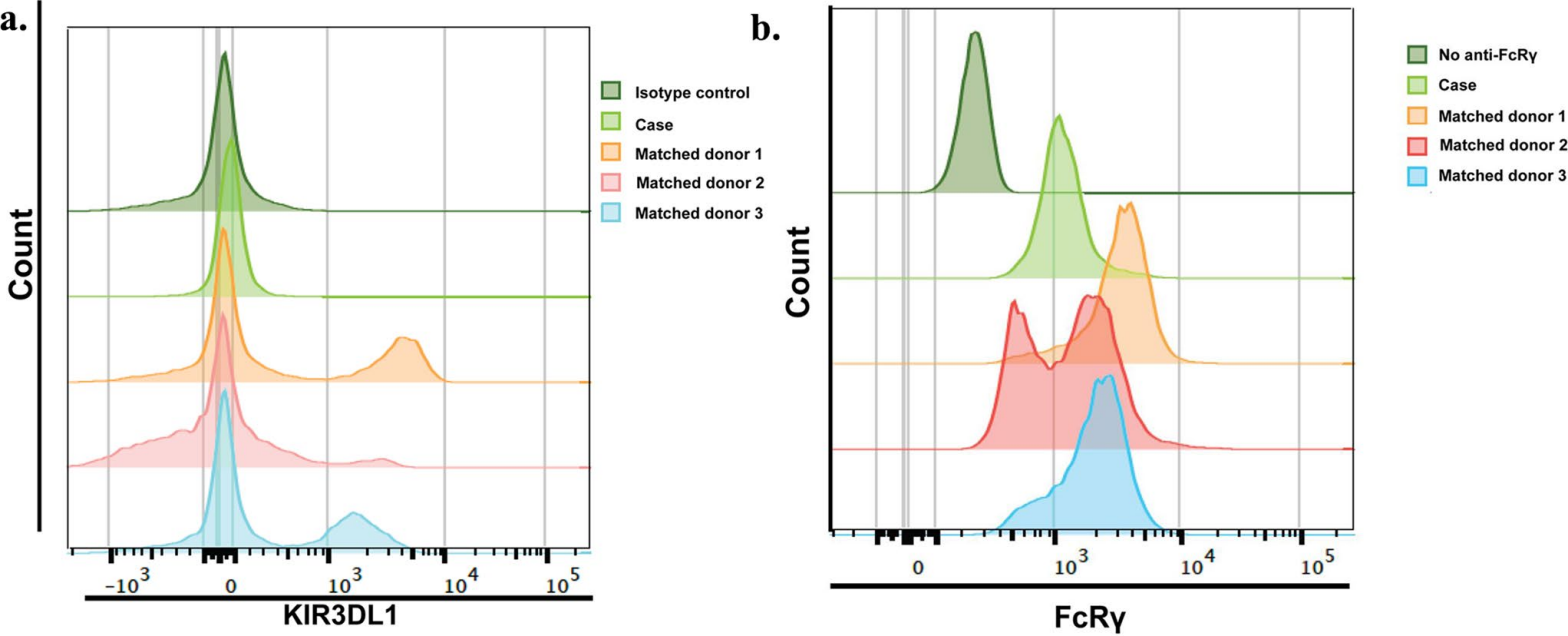
Expression of (**a**) KIR3DL1 and (**b**) FcRγ on NK cells from the case and three matched control donors. NK cells were identified as CD3^−^CD4^−^CD8^−^CD14^−^CD19-TBX-21^+^ [29]. Relative fluorescence intensities of staining with (**a**) anti-KIR3DL1 antibody and (**b**) FcRγ for the case and three matched donors are shown in the light green, orange, red, and blue histograms, as indicated. The dark green histograms represent staining with (**a**) the isotype control antibody for KIR3DL1 and (**b**) the unstained control for FcRγ (no isotype control is available because anti-FcRγ is polyclonal), which were the same for all donors. (**a**) NK cells from all of the matched control donors expressed KIR3DL1, but NK cells from the case did not. (**b**) Comparing to control donors, expression of FcRγ was 2-fold lower in the NK cells from the case

Table 3 summarizes the NK cell phenotypes in the case and the matched donors. The case and donors had similar percentages of total NK cells, by design, but in the case these cells had significantly lower percentages of CD56^bright^, KIR3DL1^+^, and CD57^+^ cells, and higher percentages of NKG2D^+^ and CD2^+^ cells. Among subsets of NK cells defined by differential intensity of CD56 expression, NKG2D and CD2 were higher on CD56^dim^ cells from the case than those from the control donors, and KIR2DL2, KIR3DL1, and CD57 were lower on CD56^dim^ cells from the case. Notably, the case’s NK cells did not express KIR3DL1 (Table 3; Fig. 3a). Intensity of FcRγ expression in NK cells of the case was significantly lower than that in the control donors (median fluorescence intensity (interquartile range): 1162 (87) vs. 2340 (1558); Fig. 3b). Percentages of memory-like or adaptive NK cells (i.e., with the phenotypes CD56^dim^NKG2C^+^NKG2A^−^, CD56^dim^CD57^+^CD2^+^FcRγ^low/−^ or CD56^dim^NKG2C^+^CD57^+^CD2^+^FcRγ^low/−^) did not differ significantly between the case and the donors. Percentages of early-differentiated NK cells (CD56^dim^NKG2A^+^NKG2D^+^CD94^+^CD57^−^KIR2DL1^−^ KIR2DL2^−^ KIR3DL1^−^) were significantly higher in the case than the control donors.

**Table 3:** Summary of expression of NK cell markers (percentages of positive cells) in total NK cells and NK cell subsets (CD56^bright^, CD56^dim^, and CD56^neg^), and percentages of adaptive and EBV-reactive NK cells of the case and matched controls

	Case	Controls								
Total NK^a^ (%)	3.0 (2.7)^b^	3.2 (3.0)								
Total NK^c^ (cells/µL)	100.5 (73.8)	116.2 (99.9)								
CD56^bright^	3.0 (1.2)^d^	**9.2 (12.7)**								
CD56^dim^	82.2 (14.7)	85.4 (21.5)								
CD56^neg^	12.9 (11.9)	5.7 (5.0)								
Staining of NK Cells for markers:										
Reported to be preferentially expressed on CD56^bright^										
	**CD94**		**NKG2A**		**NKG2D**		**CD2**		**TCF-1** ^**e**^	
	**Case**	**Controls**	**Case**	**Controls**	**Case**	**Controls**	**Case**	**Controls**	**Case**	**Controls**
Total	80.1 (3.4)	72.9 (12.7)	57.5 (8.0)	57.8 (13.7)	**85.7 (11.3)**	**66.2 (20.8)**	**86.9 (2.7)**	**77.3 (10.9)**	20.8 (78.7)	15.0 (35.0)
CD56^bright^	99.0 (1.9)	99.4 (0.8)	89.7 (7.1)	89.9 (13.2)	90.9 (8.2)	83.4 (23.8)	**94.5 (1.9)**	**92.4 (1.2)**	28.3 (95.3)	19.0 (76.8)
CD56^dim^	81.2 (3.6)	70.3 (17.0)	66.3 (13.1)	53.8 (15.4)	**91.8 (17.0)**	**66.7 (20.4)**	**89.5 (1.1)**	**76.5 (9.9)**	18.5 (83.5)	10.9 (51.9)
CD56^neg^	87.5 (9.9)	80.0 (37.4)	36.9 (40.6)	16.7 (21.5)	72.3 (16.5)	58.2 (35.8)	**76.0 (4.1)**	**40.2 (29.6)**	25.9 (80.4)	21.5 (54.4)
Reported to be preferentially expressed on CD56^dim^										
	**KIR2DL1**		**KIR2DL2**		**KIR3DL1**		**CD57**			
	**Case**	**Controls**	**Case**	**Controls**	**Case**	**Controls**	**Case**	**Controls**		
Total	17.9 (5.3)	12.4 (11.5)	16.0 (2.7)	30.8 (11.0)	**0.0 (0.2)**	**24.9 (23.3)**	**41.0 (6.7)**	**63.6 (22.4)**		
CD56^bright^	1.1 (1.2)	1.8 (2.6)	**1.6 (0.6)**	**6.2 (5.0)**	**0.0 (0.0)**	**5.1 (18.0)**	11.3 (21.8)	22.7 (19.5)		
CD56^dim^	20.9 (3.5)	14.2 (11.6)	**18.4 (2.1)**	**36.0 (18.7)**	**0.0 (0.2)**	**24.3 (26.4)**	**44.7 (5.9)**	**71.5 (15.9)**		
CD56^neg^	11.8 (7.8)	11.7 (6.8)	11.0 (2.1)	22.1 (7.7)	**0.0 (0.6)**	**11.7 (14.8)**	27.8 (10.7)	43.3 (26.1)		
To identify memory-like or adaptive NK cells^f^ and early-differentiated NK cells										
**NKG2A-NKG2C+**		**CD56** ^**dim**^ **CD57** ^**+**^ **CD2** ^**+**^ **FcRγ** ^**low/−**^		**CD56** ^**dim**^ **CD57 + CD2 + FcRγ** ^**low/−**^ **NKG2C+**			**early-differentiated NK cells**			
Case	Controls	Case	Controls	Case	Controls		Case	Controls		
3.7 (6.0)	9.3 (11.6)	24.1 (7.2)	9.0 (11.8)	2.8 (1.0)	0.5 (3.6)		21.9 **(5.5)**	**7.9 (6.2)**		

^a^ Percentages of total NK cells (CD3^−^CD4^−^CD8^−^CD14^−^CD19-TBX-21^+^) among live lymphocytes (gated based on FSC and SSC, and no staining of near-IR LIVE/DEAD™ Fixable dye). ^b^ Median (interquartile range) of data compiled from three experiments

^c^ Numbers of total NK cells were calculated by multiplying percentages of total NK cells by percentage of lymphocytes and absolute white blood count. ^d^**Bold** values indicate that the difference between the case and control donors was significant (*p* <.05). ^e^ The interquartile range of TCF-1+% cells was large because different clones of anti-TCF-1 were used in different experiments. ^f^ Percentages of mermory-like or adaptive NK cells among total NK cells measured by three phenotypes

**Fig. 4:**
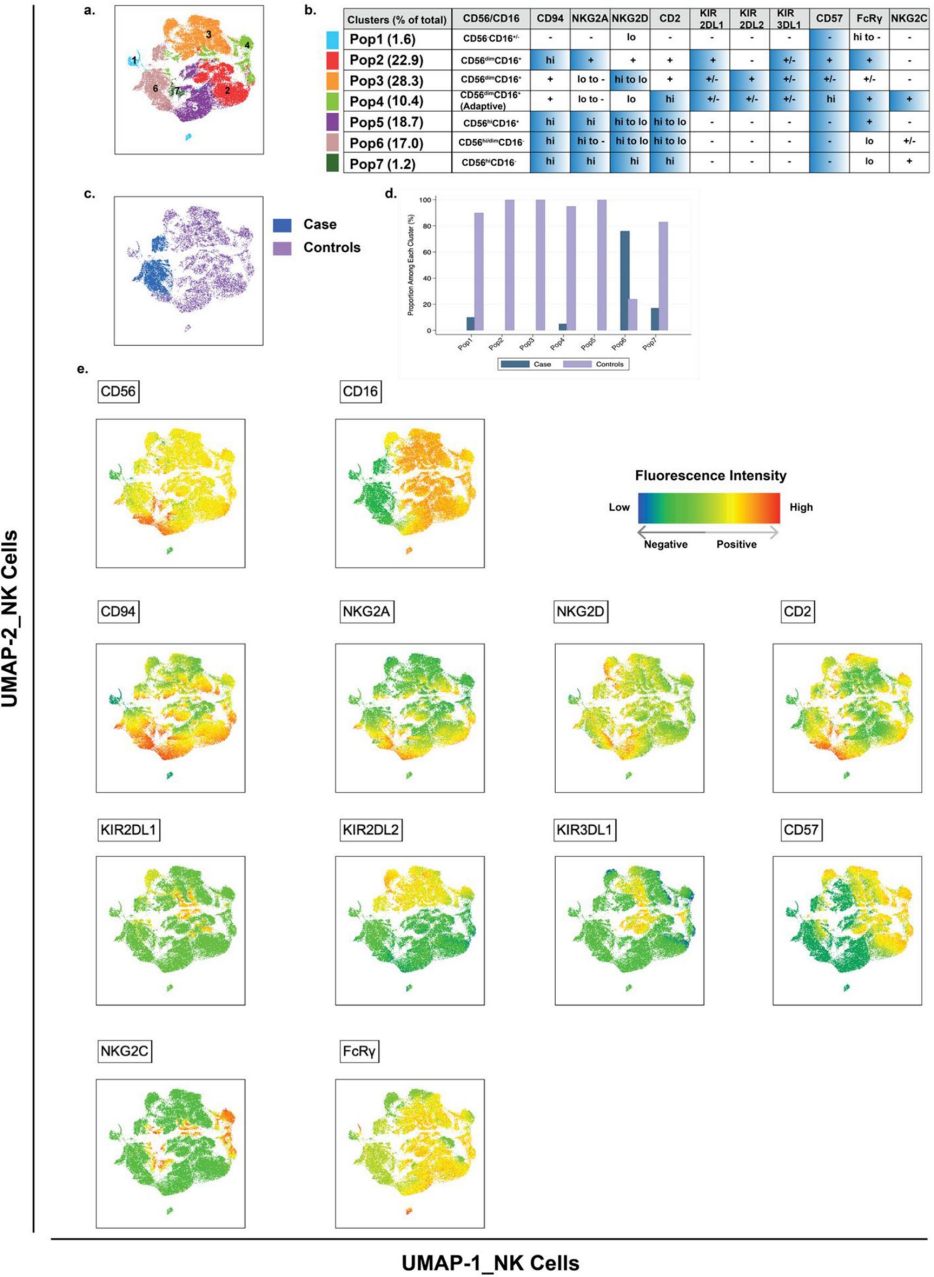
Immune phenotyping of NK cells from the case and 3 matched control donors. Frozen PBMC from the case and three matched control donors were analyzed for expression of NK cell markers. NK cells were identified as CD3^−^CD4^−^CD8^−^CD14^−^CD19-TBX-21^+^ cells (not shown). NK cell populations from the case and the matched control donors were concatenated together for dimension-reduction by UMAP and clustering by FlowSom. (**a**). UMAP displays the 7 clusters (pop 1–7) of NK cells identified by FlowSom. (**b**). The table lists the relative expression intensity of each marker on each cluster. The highlighted markers in each row, as well as CD56 and CD16, were assigned by marker enrichment modeling to define each cluster [43]. (**c**). The same UMAP shows that NK cells from the case (blue) were highly enriched in cluster 6 (Pop 6) as compared to NK cells from the matched control donors (purple). (**d**). The relative abundance of NK cells from the case (blue) and the matched control donors (purple) in each cluster. (**e**). The fluorescence intensity of each marker measured on NK cells is shown on UMAP axes. The color ranges from blue (low fluorescence intensity) to red (high fluorescence intensity)

Using dimensionality reduction and clustering analysis, 7 clusters of NK cells were identified (Fig. 4a). NK cells of the case were located primarily in cluster 6 and were absent from the adjacent cluster 5, which contained NK cells of only the controls (Fig. 4c and d). Both clusters had relatively low or absent expression of activation and maturation markers (CD57, KIR2DL1, KIR2DL2, and KIR3DL1) and high expression of markers of immature CD56^bright^ NK cells (CD94 and NKG2A) (Fig. 4b and e) [30].

### Low NK Cytotoxicity and Absent ADCC of the Case

**Fig. 5:**
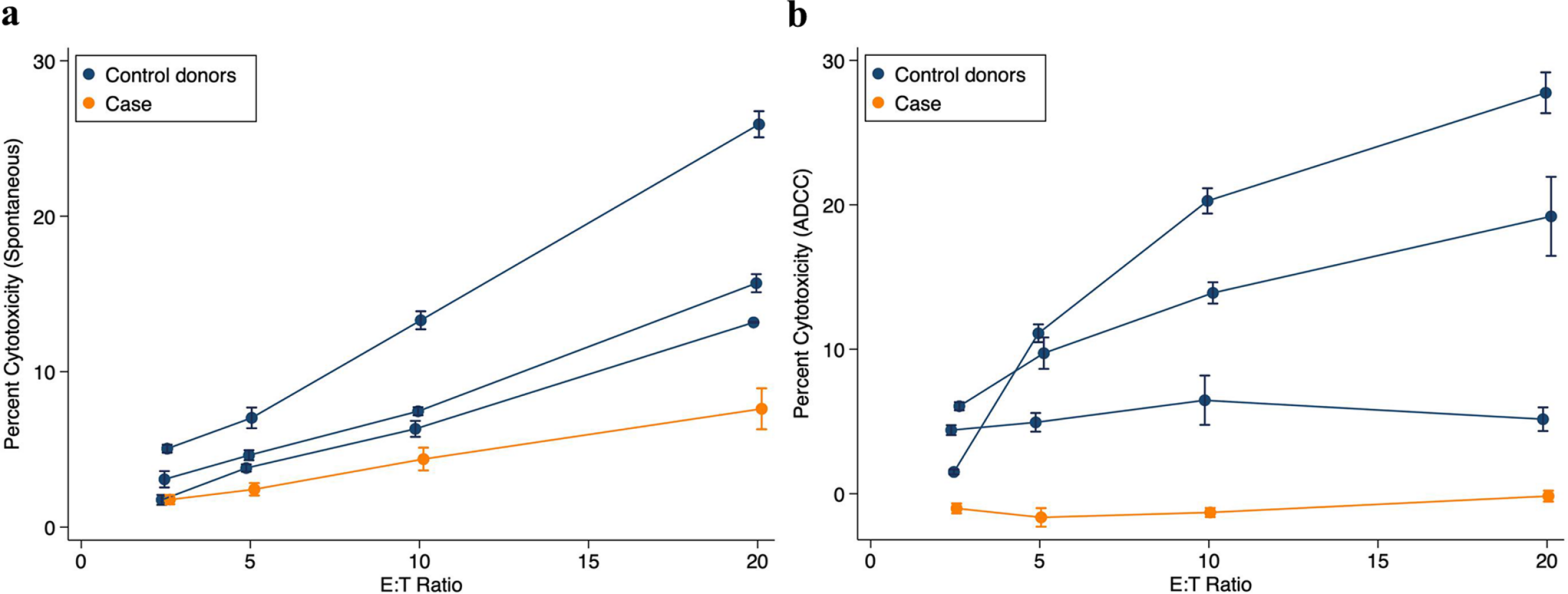
Cytotoxicity of NK cells against (**a**) K562 cells (spontaneous cytotoxicity) and (**b**) rituximab-coated Raji cells (ADCC) for the case (orange) and 3 HIV-infected men (blue) who were matched with the case by study visits, age (± 5 years), status of HIV viral suppression, and percentage of NK cells among lymphocytes. In each of three experiments, PBMC from the case and from a matched control donor were tested at the 4 Effector: Target (E: T) ratios indicated, as described in Methods. The blue circles and error bars represent the means and standard errors of triplicate measurements of cytotoxicity for the matched controls. The orange circles and error bars represent the means and standard errors of measurements of cytotoxicity for the case from the three experiments performed

NK cells from the case exhibited very low levels of cytotoxicity against K562 cells (less than 10% at the highest E: T ratio, and at all E: T ratios measured about 3-fold lower than NK cells from the matched donors (estimated using the multilevel mixed effect model), Fig. 5a; *p* <.0001). ADCC against rituximab-coated Raji cells was completely absent in the case, but substantial in the matched donors (Fig. 5b; *p* <.0001).

### Cytokine Production by NK Cells from the Case and Matched Controls

**Fig. 6:**
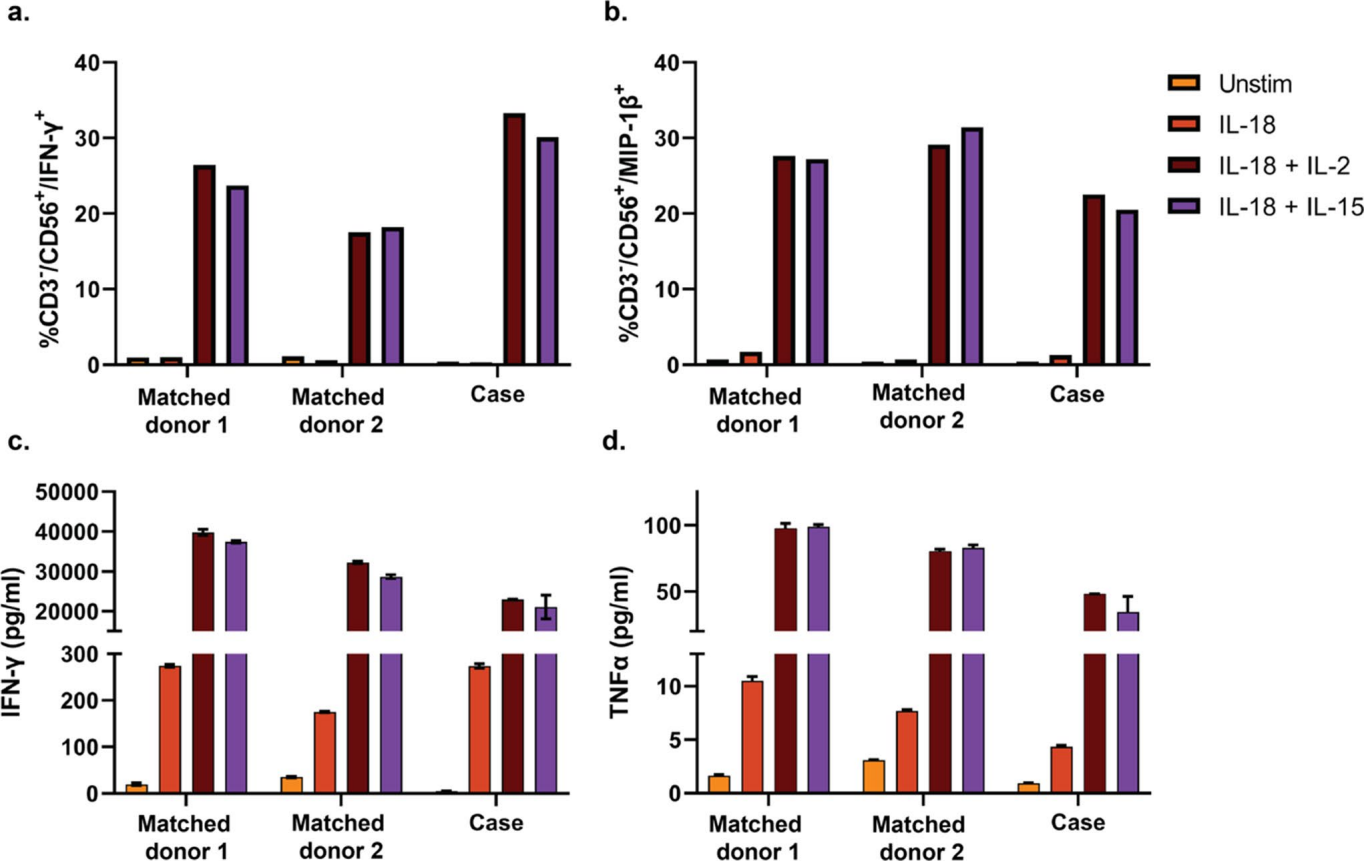
Cytokine production by NK cells from the case and two matched donors in response to innate cytokines. NK cells purified by magnetic bead negative selection were cultured in media alone (unstimulated, yellow bars), in the presence of IL-18 (red bars), IL-18 + IL-2 (burgundy bars), or IL-18 + IL-15 (purple bars) for 48 h. (**a** and **b**) Production of IFN-γ and MIP-1β by CD3^−^CD56^+^ live cells was assessed by intracellular flow cytometric analysis. (**c** and **d**) Concentrations of IFN-γ and TNF-α in the culture supernatants were assessed by multiplex electrochemiluminescence immunoassay. The error bars represent standard errors of duplicate measurements of concentrations of the cytokines in the culture supernatants

Production of IFN-γ and MIP-1β by unstimulated NK cells, and by NK cells stimulated with IL-18, IL-18 + IL-2, and IL18 + IL-15, was similar between the case and matched controls, as assessed both by flow cytometric intracellular cytokine analysis and by analysis of extracellular cytokine concentrations (Fig. 6a-c). Extracellular production of TNF-α by NK cells in response to stimulation with innate cytokines was slightly lower in the case than in the controls (Fig. 6d).

### Phenotypic and Functional Analysis of Monocytes

Because CD16A was absent on the case’s cells, monocyte subsets were identified by expression of CD14 and CD89: classical (CD14^+^CD89^+^), intermediate (CD14^dim^CD89^dim^), and nonclassical (CD14^−^CD89^−^) (Figs. S4 and S5, see details in Supplementary Material). Despite the absence of CD16, all of the monocyte subsets were present in the case (Fig. S6b). Expression of chemokine receptors (CCR2, CCR3, CX3CR1, and CXCR2), CD123 and CD163 on monocyte subsets was similar between the case and the matched donors (Supplementary Table 4). The only difference was in expression of slan on nonclassical and intermediate monocytes: percentages and numbers of slan^+^ cells were higher in the case than the controls (*p* <.05 for percentages of slan + cells, Supplementary Table 4, part C, Fig. S7).

Unstimulated monocytes from the case produced slightly higher levels of IL-1β, TNF-α, IL-6, and IL-10 than those from the control donors (Fig. S8). After stimulation in the presence of IFN-γ by a TLR-4 agonist (LPS) and a TLR-7 agοnist (CL307), monocytes from the case and those from matched donors produced similar levels of the cytokines listed above (Fig. S8). In general, monocytes produced much more of these cytokines after stimulation with the TLR-4 agonist than with the TLR-7 agonist (Fig. S8).

The case’s serum concentrations of soluble CD14 (sCD14) and sCD163 were within the range observed in the virologically suppressed HIV + men in the MACS, although his sCD14 concentration was relatively high (88th percentile) and his sCD163 was relatively low (16th percentile, Supplementary Table 5).

### Phenotypic and Functional Analysis of DCs

The case’s PBMC contained all reported subsets of peripheral DC, including plasmacytoid DC (pDC, CD123^+^CD303^+^CD11c^−^), conventional DC1(cDC1, CD123^−^CD11c^+^CD141^+^CD1c^−^), and cDC2 (CD123^−^CD11c^+^CD141^−^CD1c^+^), which could be further divided into CD5^+^CD163^−^, CD5^−^CD14^−^CD163^−^, CD5^−^CD14^−^CD163^+^, and CD5^−^CD14^+^CD163^+^ cells (Fig. S6 c-g). Percentages of DC subsets were similar between the case and the matched donors, except for a slightly higher percentage of CCR2^+^ cells among pDC, and a lower percentage of CD5^−^CD14^+^CD163^+^ and higher percentage of CD5^−^CD14^-^CD163^+^ cells among cDC2 (Supplementary Table 6).

Monocyte-derived mature DCs from the case and matched donors had greater expression of CD83, CD86, and Siglec-1 than immature DCs; the degree of upregulation of these markers was higher in the case than the controls (Fig. S9). The case’s monocyte-derived mature DCs produced similar levels of IL-12p70, IL-10, IL-6, IL-8, and TNF-α as those from the matched controls (Fig. S10).

### Status of Herpesvirus Infections in the Case

The case was seropositive for most of the herpesviruses tested, except HHV-6 and HHV-7 (Table 4). DNA of CMV, HHV-6, EBV, and HHV-8 was undetectable in the peripheral blood plasma of the case in samples obtained from 2004 to 2009.

**Table 4:** Results of testing of the case for herpesvirus infections

Virus	Serostatus (IgG)	Year of serostatus test	Viral DNA (in plasma)
HSV-1	+	2010	NA^a^
HSV-2	+	2010	NA
VSV	+	2010	NA
CMV	+	2004	Undetectable
HHV-6	-	2010	Undetectable
HHV-7	-	2010	NA
EBV	+	2004	Undetectable
HHV-8	+	2007	Undetectable

^a^ Not available

### Percentages of NKT-like Cells in the Case

Percentages of NKT-like cells (CD3^+^CD56^+^/CD16^+^) among lymphocytes of the case were consistently higher than those of the control donors across two years of study (Fig. S11).

## Discussion

This study investigated a person whose NK cells and monocytes did not express CD16A on initial flow cytometric analysis. The complete absence of CD16A expression was confirmed using two anti-CD16A antibodies that detect different epitopes of CD16A. Further, we found that the case had compound heterozygous deletion of the CD16A-encoding *FCGR3A* gene that would preclude production of CD16A. To our knowledge, a copy number of 0 for *FCGR3A* has been reported only once, in 2 out of 17,754 people tested (0.01%) [44]. However, that study used an indirect method, single nucleotide polymorphism analysis, to determine copy number, and did not validate their results using other methods [44]. CD16A is essential for NK-cell-mediated spontaneous cytotoxicity and ADCC [5, 6, 18, 45], and the present case’s NK cells exhibited greatly decreased spontaneous cytotoxicity and no detectable ADCC. Consistent with no ADCC, the serum concentration of IgG1 of the case was slightly higher than the normal range as CD16A mainly binds to IgG1 for ADCC [46].

Compared to the controls, the case had significantly lower percentages of CD56^bright^ immature NK cells, higher expression of NKG2D and CD2 in CD56^dim^ NK cells, markers that are highly expressed on CD56^bright^ immature NK cells [30, 47], and lower expression of markers of maturation and differentiation on CD56^dim^ NK cells, such as KIR2DL2, KIR3DL1, and CD57. In clustering analysis, NK cells from the case were enriched in a cluster whose expression profile resembled that of immature CD56^bright^ NK cells [30]. Since maturation of NK cells has been associated with cytotoxicity [30], the lower spontaneous cytotoxicity of NK cells from the case may be related to their immaturity relative to that of the controls.

Two other factors may be contributing to the lower spontaneous cytotoxicity of the case’s NK cells. First, the case’s NK cells had low expression of KIR2DL2 and complete absence of KIR3DL1. Interactions between KIR receptors and self MHC class I molecules are important for the effector function of NK cells during maturation [48]. Second, the case’s NK cells had lower expression of FcRγ than the controls. FcRγ reportedly binds to the cytoplasmic tail of CD16A and serves as a signaling adaptor of CD16A [49], so CD16A may be important to stabilize expression of FcRγ. Deletion of FcRγ using CRISPR-Cas9 resulted in lower spontaneous cytotoxicity of NK cells [50]. These data suggest that lower expression of both KIR receptors and FcRγ in the case’s NK cells could impair cytotoxicity.

NK cell deficiency (NKD) is characterized by abnormal susceptibility to viral infections, especially with herpesviruses [51–53]. NKD are categorized into classical and functional [52, 53]. Classical NKD is defined as absence or very low levels (≤1%) of NK cells in peripheral blood, and has been linked to mutations in 6 genes [52, 54]. Functional NK cell deficiency is characterized by normal numbers but defective functions of NK cells [52, 53]; CD16A deficiency is its only known cause [5, 18]. Thus, the present case exhibited a functional NK cell deficiency.

Two types of functional CD16A deficiency have been described. The first is a homozygous point mutation (L66H) in *FCGR3A*, associated with decreased NK cell-mediated spontaneous cytotoxicity but intact ADCC [5], binding of CD16 by mAb 3G8 but not mAb B73.1 [5, 23], and clinical manifestations of frequent upper respiratory infections, recurrent HSV stomatitis, recurrent herpes whitlow, and EBV-associated Castleman’s disease [5]. The second is deletion and fusion of *FCGR3A*, with absence of ADCC, persistent EBV infection and renal failure [18]. The present case had none of these clinical manifestations.

NK cells of the case differed from those in reported cases with CD16A deficiency. Expression of CD2 was normal, rather than reduced as reported in NK cells from people with the L66H mutation in CD16 [5], although another case with complete CD16A deficiency had normal CD2 expression [18]. However, the latter case did not manifest the less mature profile, the decreased spontaneous cytotoxicity, the low expression of FcRγ, and the complete lack of KIR3DL1 expression of NK cells seen in the present case [18]. It is possible that CD16A and KIR3DL1 act together in the development and differentiation of NK cells. This possibility merits further study. The percentages of memory-like or adaptive NK cells in the present case were comparable to those in the controls, suggesting that factors other than CD16A could affect NK cell differentiation, such as CMV infection and chronic HIV infection [55, 56]. Both the present case and the reported case of complete CD16A deficiency were seropositive for CMV infection, but different CMV isolates and levels of CMV-specific antibodies could affect percentages of adaptive NK cells [56].

Because the case did not express CD16A on monocytes, we developed and validated a strategy to identify classical, intermediate, and nonclassical monocytes, based on expression of CD14 and CD89 rather than expression of CD14 and CD16. The case did not show any defect in the development of monocyte subsets or in the in vitro function of these cells. Percentages of DC subsets were also similar between the case and matched control donors. These results suggest that the case’s monocytes and DCs were not affected by his CD16A deficiency.

It is unclear why the present case showed no evidence of the susceptibility to herpesvirus infections reported in other people with NKD [52, 53]. Most such people had severe infections during their childhood [5, 18, 54, 57], but the case did not recall any serious childhood (or adult) illnesses or hospitalizations. Lack of exposure is unlikely to account for this, because the case had antibodies to several human herpesviruses (CMV, EBV, HHV-8, VSV, HSV-1 and HSV-2). Other populations have been reported to have very low levels (median: 45/µl) of NK cells without experiencing severe viral infections, such as long-term survivors (up to 30 years) of allogeneic hematopoietic stem cell transplantation or people undergoing gene therapy (for common cytokine γ chain deficiency only) for severe combined immunodeficiency [58, 59]. Allelic polymorphisms in the T cell receptor may provide increased affinity and function of T cells against viral infection [60]. However, multiple polymorphisms would be required to confer protective T cell responses against multiple herpes viruses. It is also possible that NK cells from the case perform immunoregulatory functions that prevent severe herpesvirus infections. For example, CD56^bright^ NK cells produce immunoregulatory cytokines, such as IFN-γ, TNF-α, and IL-10, that may support viral clearance and reduce inflammation associated with severe viral infections [61]. In addition, CD56^dim^ NK cells can respond to innate cytokines (i.e., IL-18, IL-12 and IL-15) to facilitate DC maturation and type-1 polarization and viral clearance by producing IFN-γ [55, 35, 36, 62]. To support this notion, NK cells from the case produced similar amounts of cytokines in response to innate cytokines as the matched donors. Another possibility is that the case’s monocytes and DCs may be prone to enhanced responsiveness to infections. This possibility is supported by (a) higher expression of slan on nonclassical monocytes, which suggests higher responsiveness to GM-CSF [63]; (b) higher concentrations of cytokines produced by the monocytes without any stimulation; (c) higher percentage of CCR2^+^ pDC, which could be recruited to inflamed regions by CCL2 [64]; and (d) greater upregulation of co-stimulatory receptors and Siglec-1 on monocyte-derived DCs in response to maturation factors. An additional possibility is that the case’s NKT and NKT-like cells compensate for the NK cell cytolytic deficiency. NKT cells have been associated with control of herpesvirus infections in mouse models [65], and two case reports have described disseminated VSV infection in humans with very low percentages of NKT-like cells (< 0.03%) [66, 67].

The two deletions of *FCGR3A* in the present case were probably inherited, as in the previous case with complete CD16A deficiency [17]. However, it was impossible to confirm this as both parents of the case were deceased when the case was identified. It is interesting that having a copy number of 0 has been reported much more frequently for *FCGR3B* than for *FCGR3A*, although the deletions of both genes are mediated by the same mechanism and having only 1 copy of either gene has been associated with immunological diseases, such as rheumatoid arthritis, systemic lupus erythematosus, and anti-glomerular basement membrane antibody disease [9, 10, 12, 68–71]. Considering the importance of *FCGR3A*, but not of *FCGR3B*, for the function of NK cells, it is possible that deletion of *FCGR3A* has been negatively selected during evolution.

In summary, the case studied exhibited: (1) absence of CD16A expression caused by compound heterozygous deletion of *FCGR3A*; (2) absent ADCC and very low spontaneous cytotoxicity by NK cells, despite normal abundance of these cells; (3) a lack of expression of KIR3DL1 by NK cells, and (4) impaired maturation of NK cells, possibly due to lack of expression of both CD16A and KIR3DL1, but intact formation of memory-like/adaptive NK cells. These defects were not accompanied by increased susceptibility to herpesvirus infections, suggesting that NK cell deficiency does not necessarily lead to clinical immunodeficiency and that other immune cells, such as T cells, B cells, monocytes and DCs, may sometimes compensate for deficient NK cell function. It is also possible that even a very low level of immunoregulatory functioning of NK cells may provide adequate protection against these infections. These findings may lead to better treatment and/or prevention of infections with viruses such as herpes viruses.

## Electronic Supplementary Material

Below is the link to the electronic supplementary material.

Supplementary Material 1

## Data Availability

No datasets were generated or analysed during the current study.
