# Supplementary material for: Complete CD16A Deficiency and Defective NK Cell Function in a Man Living with HIV
Source: J Clin Immunol. 2025 May 24;45(1):98. doi: 10.1007/s10875-025-01886-y (PMC12103316; doi:10.1007/s10875-025-01886-y)
Supplement: Supplementary file 1 — Supplementary Material 1 [file 10875_2025_1886_MOESM1_ESM.docx]

Supplementary Material

Complete CD16A deficiency and defective NK cell function in a man living with HIV

Submitted to Journal of Clinical Immunology

Weiying Zhang^1^, Alan F. Scott^2^, David W. Mohr^2^, Roxann Ingersoll^2^, Peter E. Shoucair^3^, Jay H. Bream^1, 4^, Tricia L. Nilles^1^, Hao Zhang^1^, Yue Chen ^3^, Robbie B. Mailliard^3^, Joseph B. Margolick^1^

^1^ Department of Molecular Microbiology and Immunology, Johns Hopkins Bloomberg School of Public Health, Baltimore, Maryland, USA.

^2^ Department of Genetic Medicine, Johns Hopkins School of Medicine, Baltimore, Maryland, USA.

^3^ Department of Medicine, University of Pittsburgh School of Medicine, Pittsburgh, PA, USA.

^4^ Graduate Program in Immunology, Johns Hopkins School of Medicine, Baltimore, Maryland, USA

Address correspondence to Joseph B. Margolick, MD, PhD, Department of Molecular Microbiology and Immunology, Johns Hopkins Bloomberg School of Public Health, 615 N Wolfe St., Rm E5153, Baltimore, MD 21205.

Email: [jmargol1@jhu.edu](mailto:jmargol1@jhu.edu)

Phone: 410.955.1436

Fax: 410.955.0105


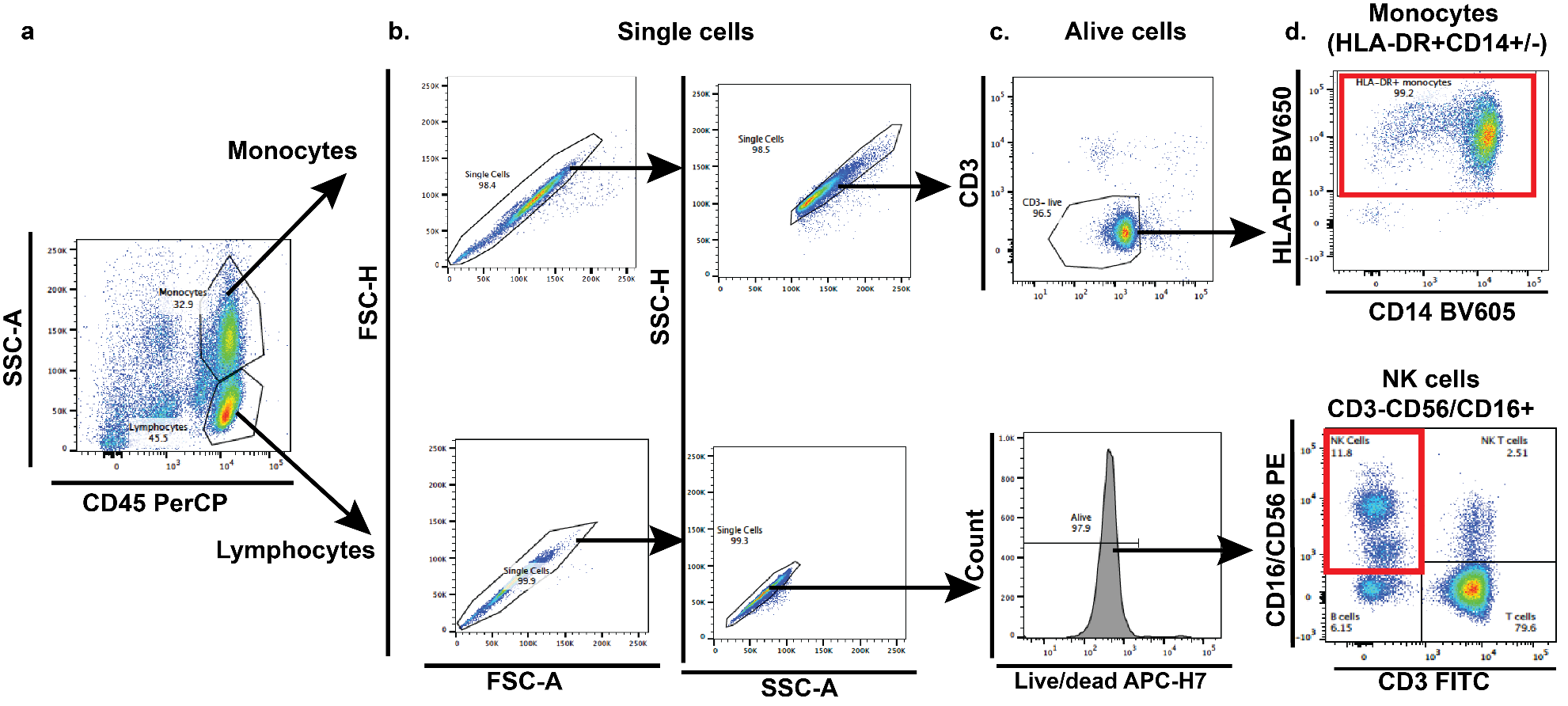


Fig. S1. Gating scheme for identifying CD16A expression on monocytes (upper panel) and NK cells (lower panel). (a) Monocytes and lymphocytes were identified by expression of CD45 and different side scatter (SSC; high for monocytes and low for lymphocytes). (b) Forward scatter (FSC)-area vs FSC-height, and SSC-area vs SSC-height were used to identify single cells. (c) lack of expression of live/dead dye (live/dead^-^) and of CD3 were used to identify live monocytes (upper panel), and live/dead- was used to identify live lymphocytes (lower panel). (d) Monocytes were further identified as HLA-DR^+^CD14^+/-^ cells (upper panel), and NK cells were identified as CD3^-^CD16/CD56^+^ cells (lower panel).


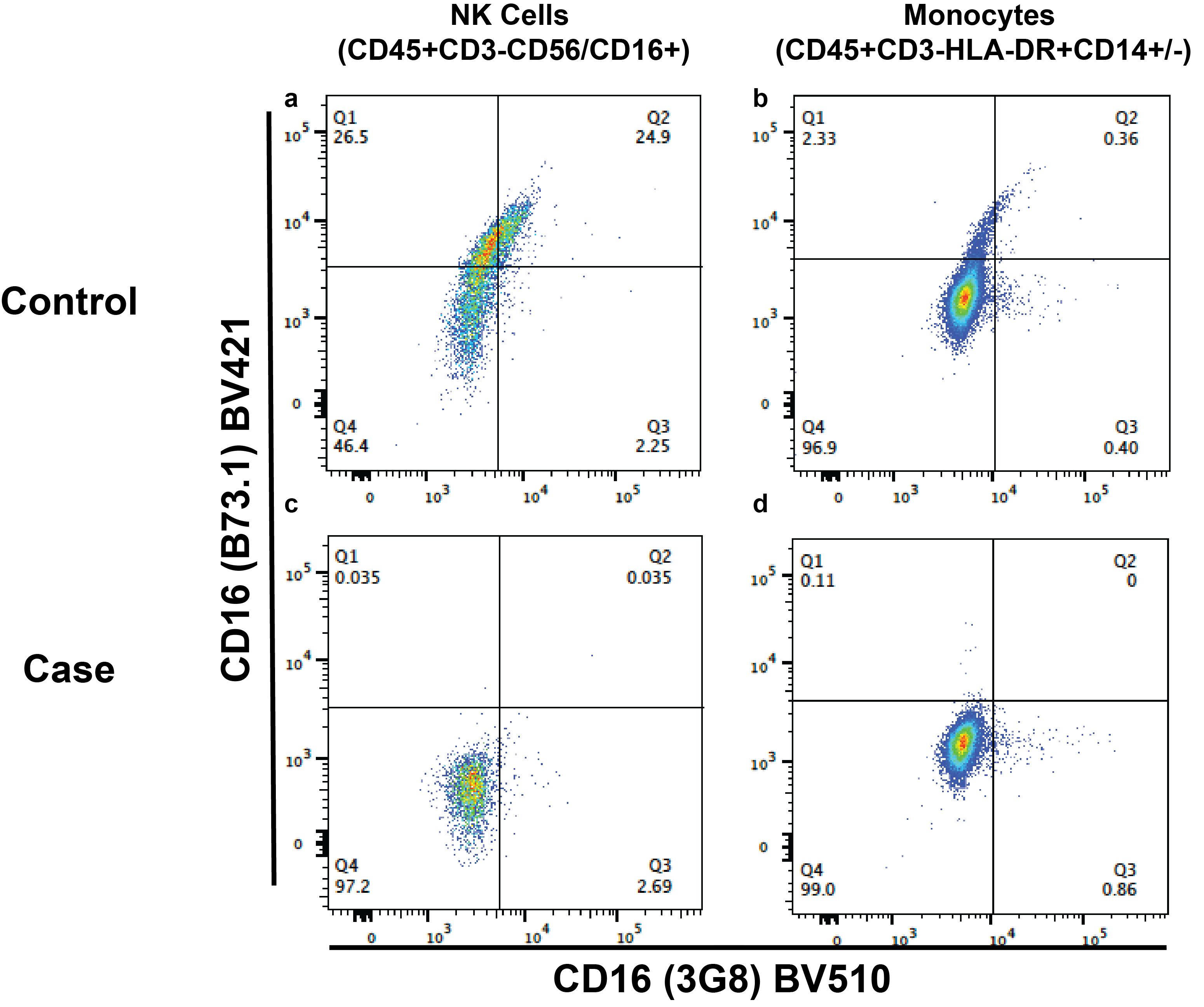


Fig. S2. Lack of intracellular expression of CD16A in NK cells and monocytes of the case. The flow cytometric plots showing the intracellular expression of CD16A on (a and c) NK cells and (b and d) monocytes of a HIV- control and the case using mAbs against 2 epitopes of CD16A (B73.1 and 3G8). NK cells and monocytes were defined as in Fig. S1. The gates for identifying CD16A+ cells were set based on the FMO shown in Fig.1. Intracellular expression of CD16A as identified by both clones of anti-CD16A antibodies (Q2) was present in cells from the control (a and b) but not from the case (c and d).


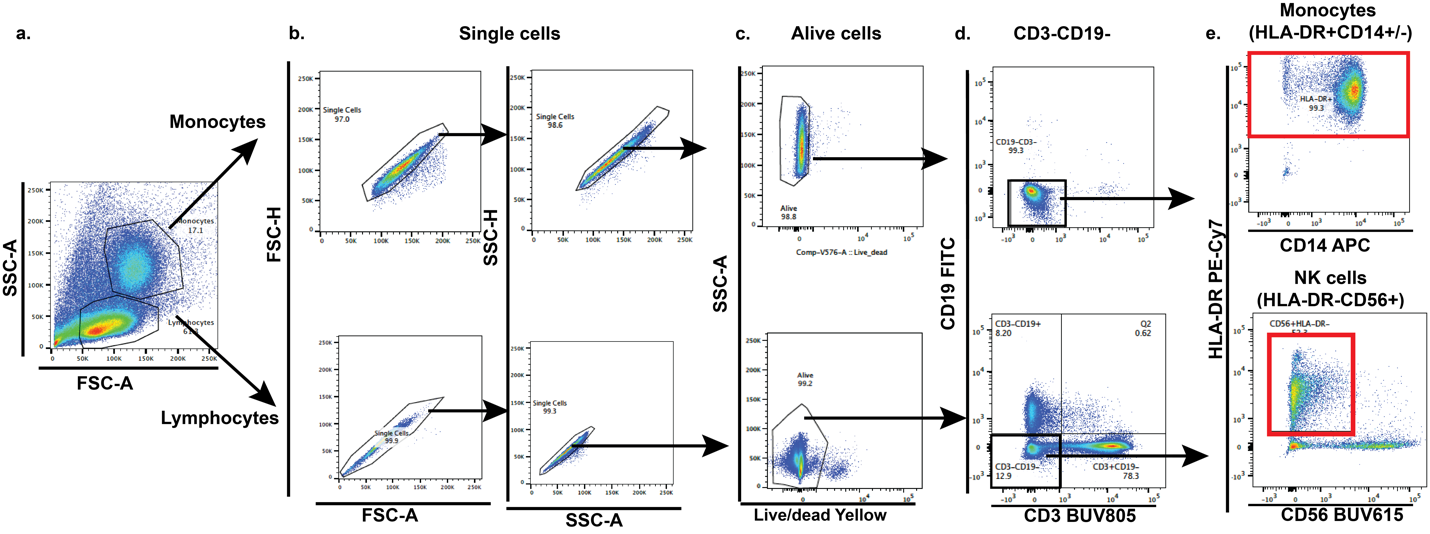


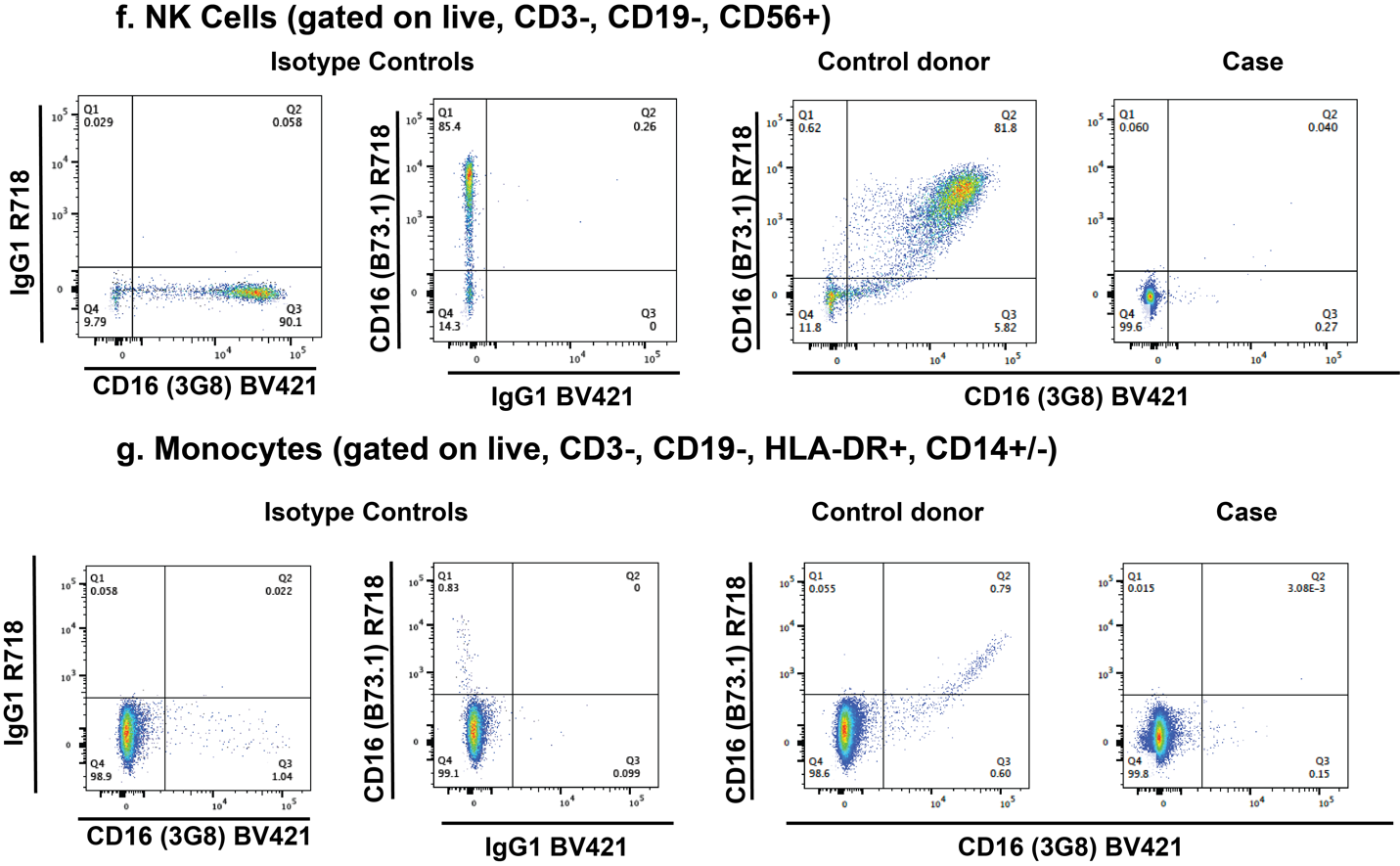


Fig. S3. Lack of expression of CD16A on NK cells and monocytes of the earliest available specimen from the case. NK cells and monocytes were identified as shown in panels a-e. (a) Total lymphocytes and monocytes were identified by different side SSC (high for monocytes and low for lymphocytes). (b) and (c) Single cells and live cells were identified as described in Fig. S1. (d) Lack of expression of CD3 and CD19 were used to identify non-T and non-B cells. (e) Monocytes were identified as HLA-DR^+^CD14^+/-^ cells (upper panel), and NK cells were identified as HLA-DR-CD56^+^ cells (lower panel). (f) and (g) The flow cytometric plots show the expression of CD16A on (f) NK cells and (g) monocytes of a virally suppressed HIV-infected donor (control donor) and the case using two monoclonal antibodies (mAbs), B73.1 and 3G8, as mentioned in Fig. 1. Gates for identifying CD16A expression were set based on staining of isotype controls (two leftmost columns). Consistent with the results shown in Fig. 1, CD16A was detected by both mAbs on the cells of the control donor (third column from the left), but not the case (rightmost column) for both NK cells and monocytes.

**Development and validation of an alternative gating strategy to identify monocyte subsets**

Based on a previous report of differential expression of markers on monocyte subsets [1], we developed a strategy to identify monocyte subsets by expression of CD14 and CD89 : classical (CD14^+^CD89^+^), intermediate (CD14^dim^CD89^dim^), and nonclassical (CD14^-^CD89^-^). In the matched donors, three monocyte subsets identified by expression of CD14 and CD89 were very similar to those identified by expression of CD14 and CD16 (Fig. S4 h and i). Furthermore, differential expression of CD123, CD163, slan, and chemokine receptors, such as CCR2, CCR3, CX3CR1, and CXCR2, on the monocyte subsets were consistent with those that have been reported [2–6]. Specifically, expression of CCR2, CCR3, CXCR2, and CD163 were higher in classical (CD14^+^CD89^+^) than the other two subsets (intermediate (CD14^dim^CD89^dim^) and nonclassical (CD14^-^CD89^-^), Supplementary Fig. S5 a-i and Supplementary Table 4). CD123 were preferentially expressed on intermediate and nonclassical monocytes (Fig. S5 g-i, and Supplementary Table 4). CX3CR1 and slan were expressed on nonclassical monocytes but not on classical monocytes (Fig. S5 a-c, j-I, and Supplementary Table 4).


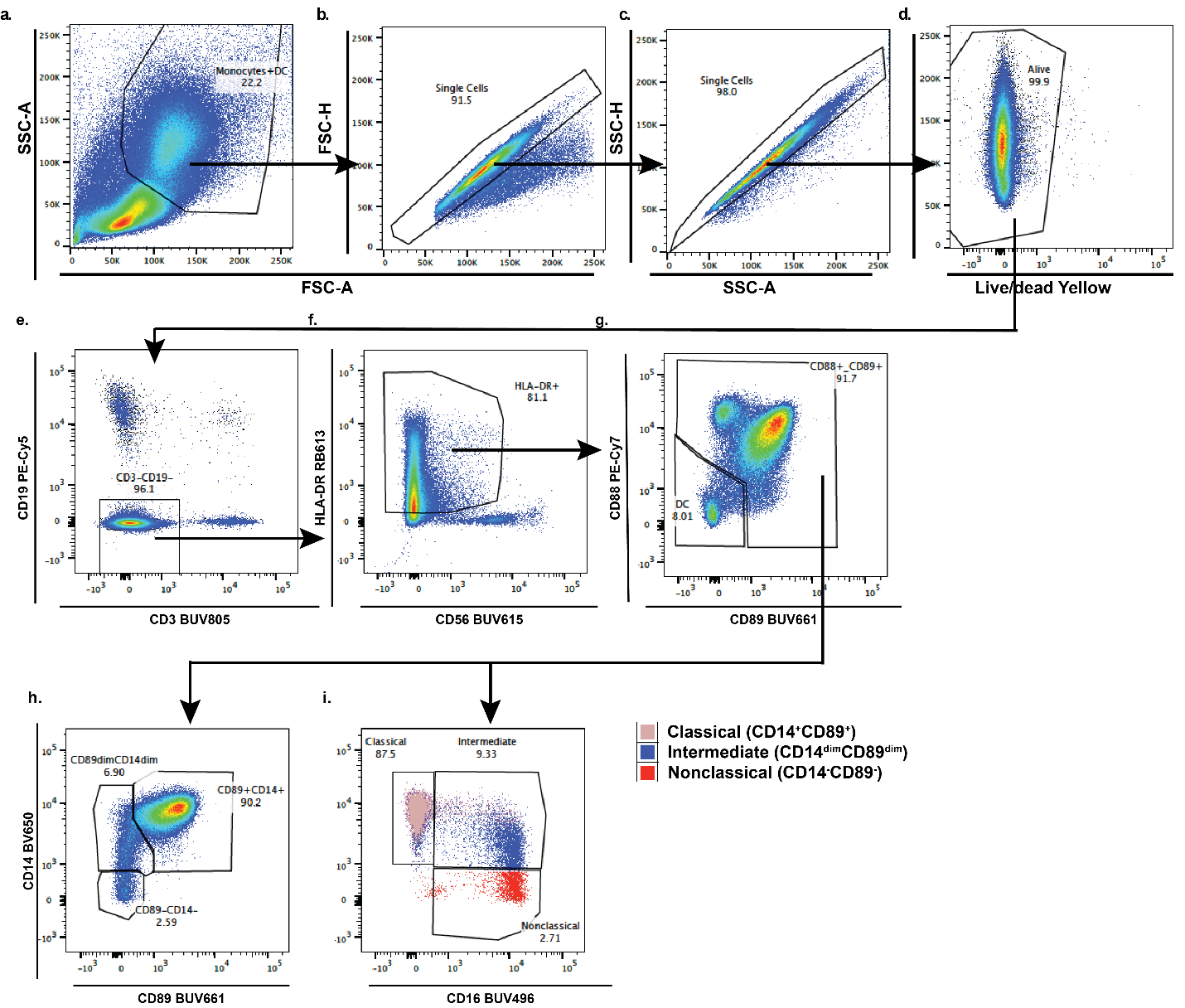


Fig. S4 Identification of monocyte subsets (classical, intermediate, and non-classical) by expression of CD14 and CD89. After gated on (a) large cells (FSC-A high and SSC-A high) (b and c) single, and (d) alive cells, total monocytes were identified as CD3^-^, CD19^-^, CD56^-^, HLA-DR^+^, CD88^+^, and CD89^+/-^ (e-g). Then they were divided into three subsets based on expression of (h) CD14 and CD89 or of (i) CD14 and CD16. In i), using monocytes from a control donor, the three subsets defined in (h) were superimposed on monocytes subsets defined by expression of CD14 and CD16, with pale pink representing classical (CD14^+^CD89^+^) in h), blue representing intermediate (CD14^dim^CD89^dim^) in h), and red representing nonclassical (CD14^-^CD89^-^) monocytes in h).


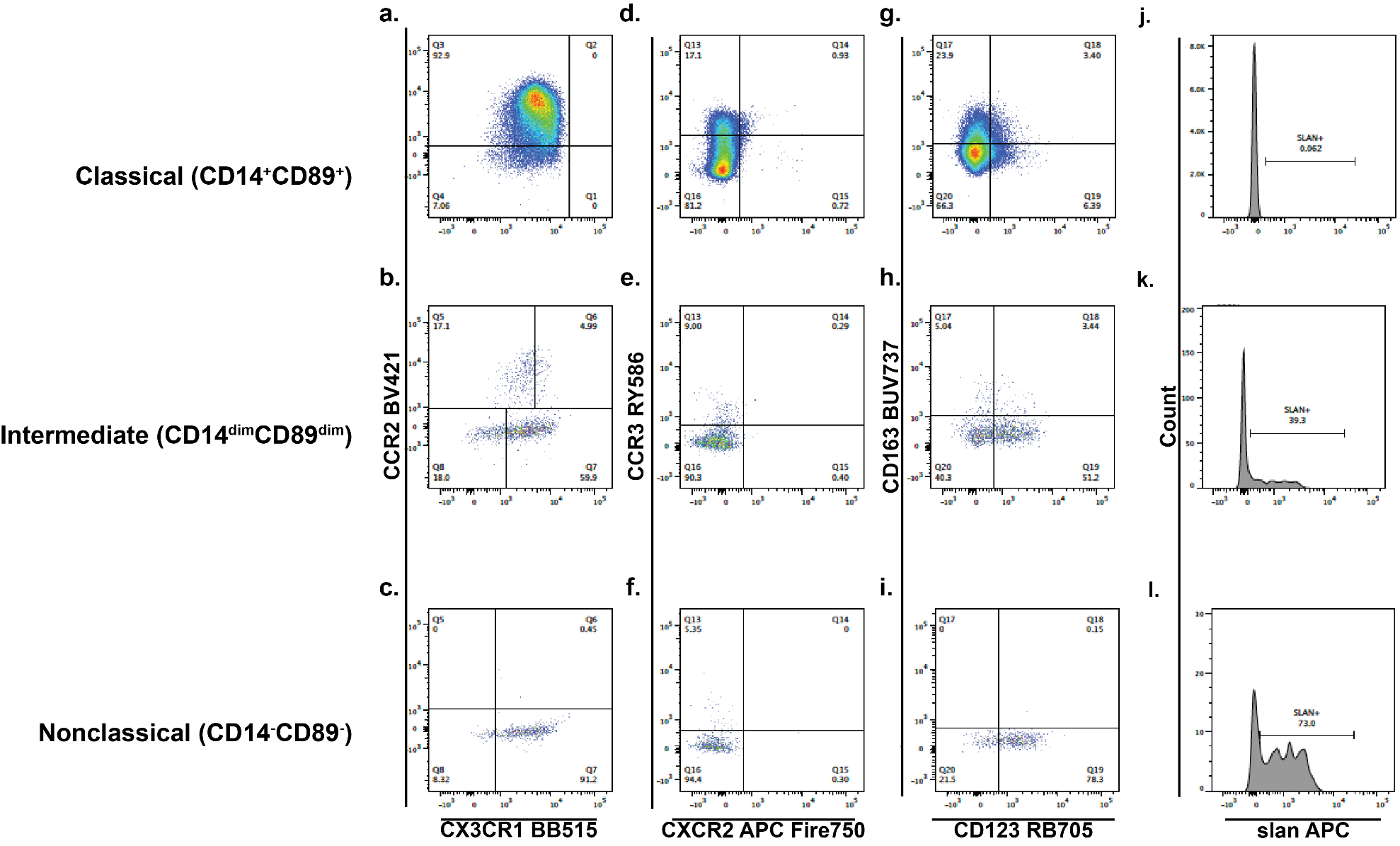
 Fig. S5. Expression of chemokine receptors (CCR2, CX3CR1, CCR3, and CXCR2), CD163, CD123, and slan on monocyte subsets of the case. The flow cytometry dot plots and histograms show the expression of (a-c) CCR2 vs CX3CR1, (d-f) CCR3 vs CXCR2, (g-i) CD163 vs CD123, and (j-l) slan on classical, intermediate, and nonclassical monocytes. a-c, The percentage of CCR2^+^ cells was higher in classical than in intermediate monocytes, and CCR2 was not expressed on nonclassical monocytes. In contrast, the percentage of CX3CR1^+^ cells was higher on nonclassical than intermediate and classical monocytes. d-f, CCR3^+^ and/or CXCR2^+^ cells were mainly present in classical monocytes. g-I, CD163 and CD123 showed the opposite trend of expression on monocyte subsets as CCR2 and CX3CR1. j-i, Slan^+^ cells were mainly present in nonclassical monocytes and absent on classical monocytes.


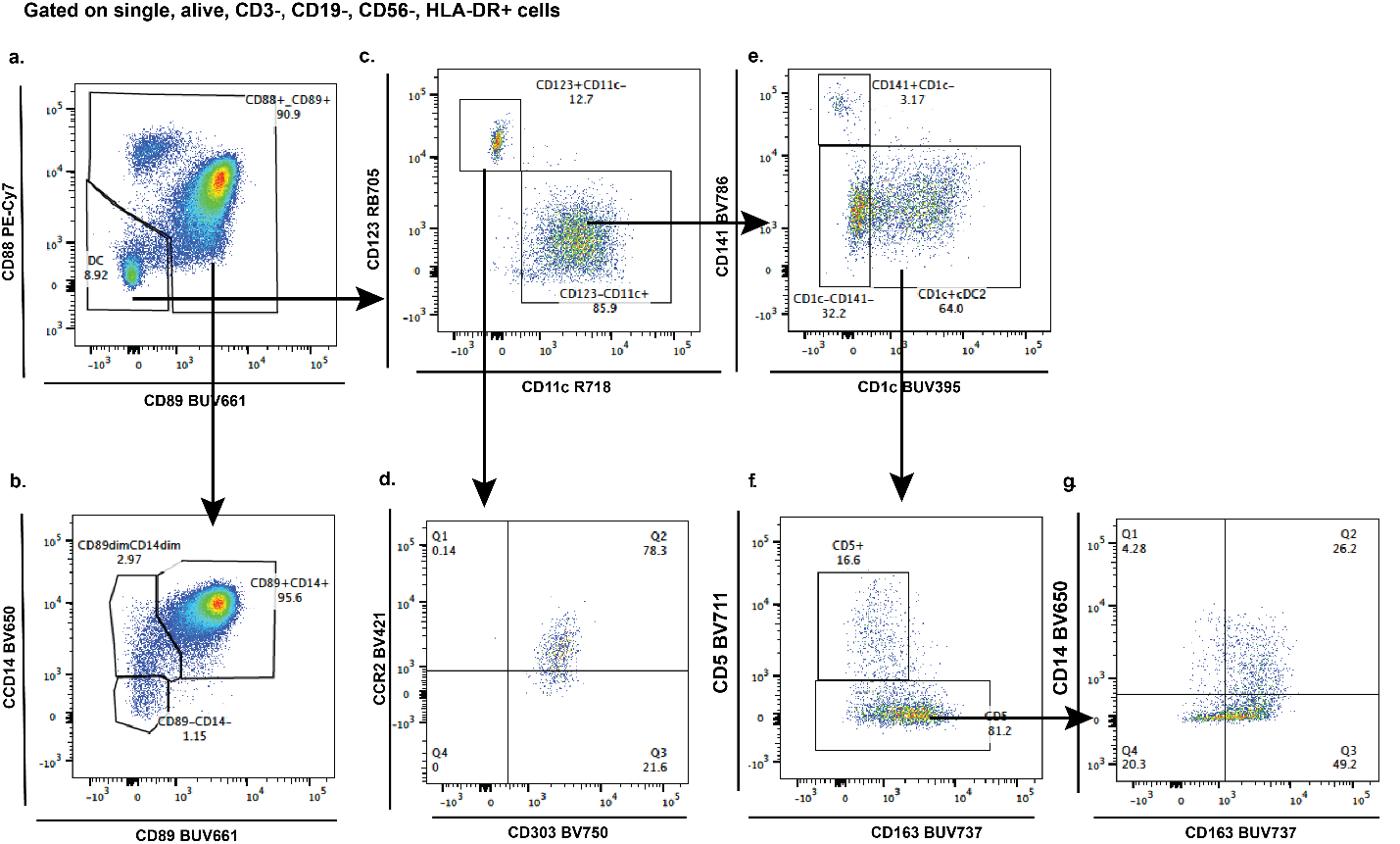


Fig. S6. Immunophenotypic analysis of monocyte and DC subsets in the PBMC of the case. (a) Total monocytes and DCs were identified as shown in Supplementary Fig.S4. (b) Total monocytes were identified as CD88+CD89+/- and divided into three subsets as described in Supplementary Fig. S4. Total DCs were divided into (c and d) pDC (CD123^+^CD303^+^CD11c^-^), (e) cDC1 (CD123^-^CD11c^+^CD141^+^CD1c^-^) and cDC2 (CD123^-^CD11c^+^CD141^-^CD1c^+^). (f and g) cDC2 were further divided into CD5^+^CD163^-^, CD5^-^CD14^-^CD163^-^, CD5^-^CD14^-^CD163^+^, and CD5^-^CD14^+^CD163^+^ cells.


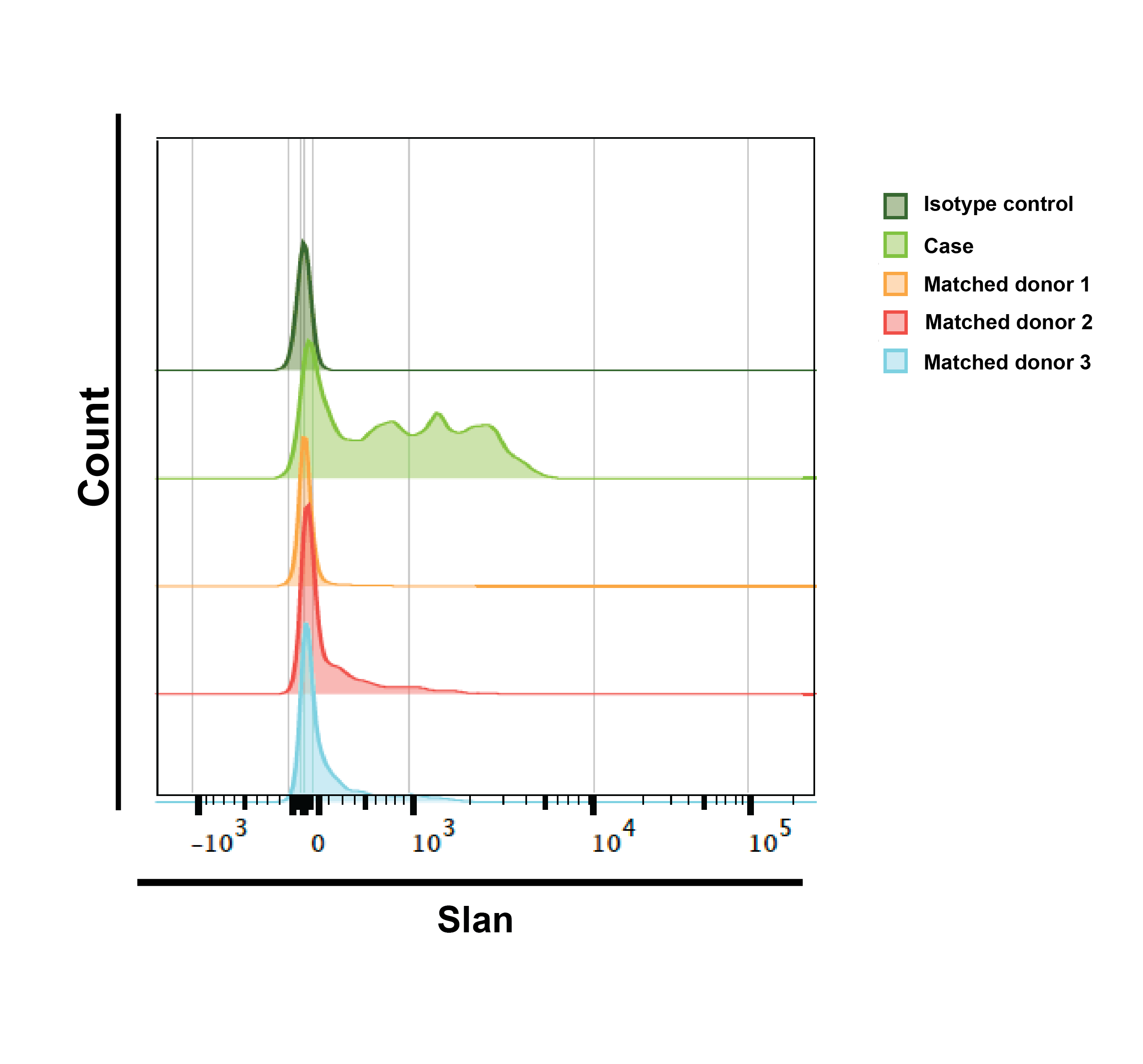


Fig S7. Expression of slan on nonclassical monocytes from the case and three matched control donors. Nonclassical monocytes were identified as CD14^-^CD89^-^. Relative fluorescence of staining with anti-slan antibody, as well as an isotype control antibody, for the case and three matched donors are shown, as indicated. The case had much higher percentage of slan^+^ nonclassical monocytes than the matched donors (73% vs 30% (12.9) (median (IQR) of matched donors)).


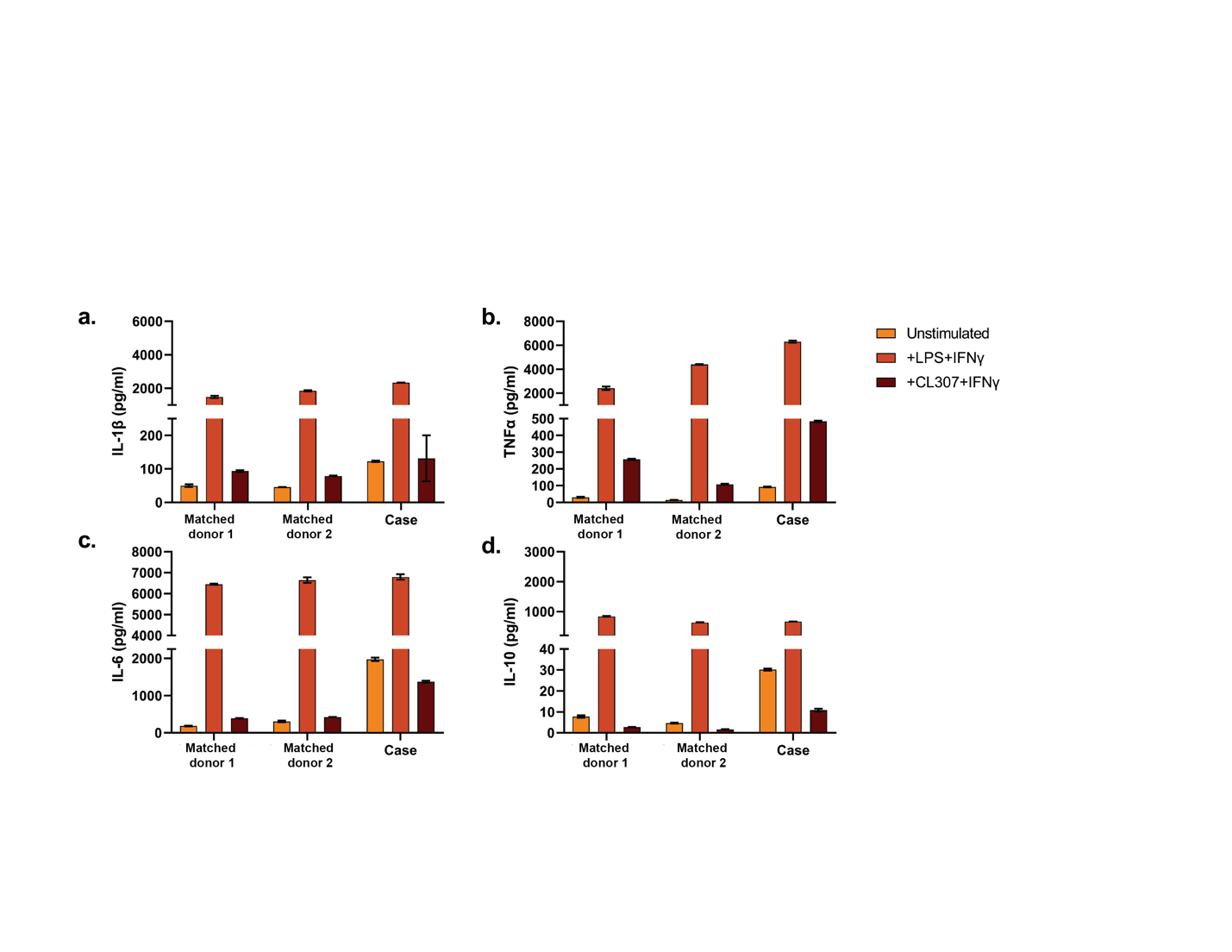


Fig. S8. Production of (a) IL-1β, (b) TNF-α, (c) IL-6, and (d) IL-10 by monocytes from the case and two matched donors. Monocytes isolated by CD14-positive magnetic beads were either unstimulated, or stimulated with LPS or CL307 in the presence of IFN-γ for 24 hours. Cytokines in the supernatants of the cell culture were measured by multiplex electrochemiluminescence immunoassay.


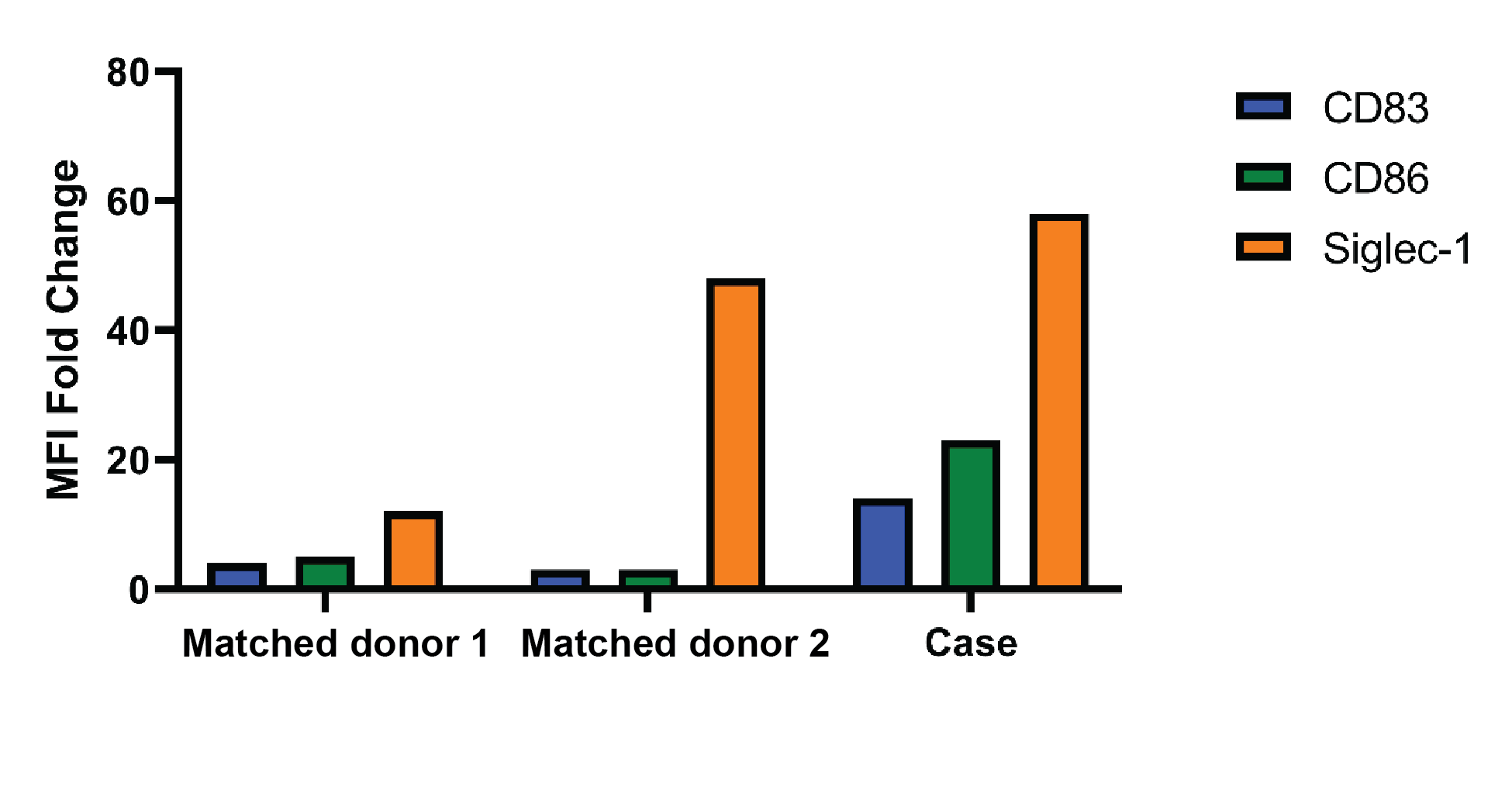


Fig. S9. Fold change of median fluorescence intensity of CD83, CD86, and Siglec-1 from immature DC to mature DC in the case and two matched donors.


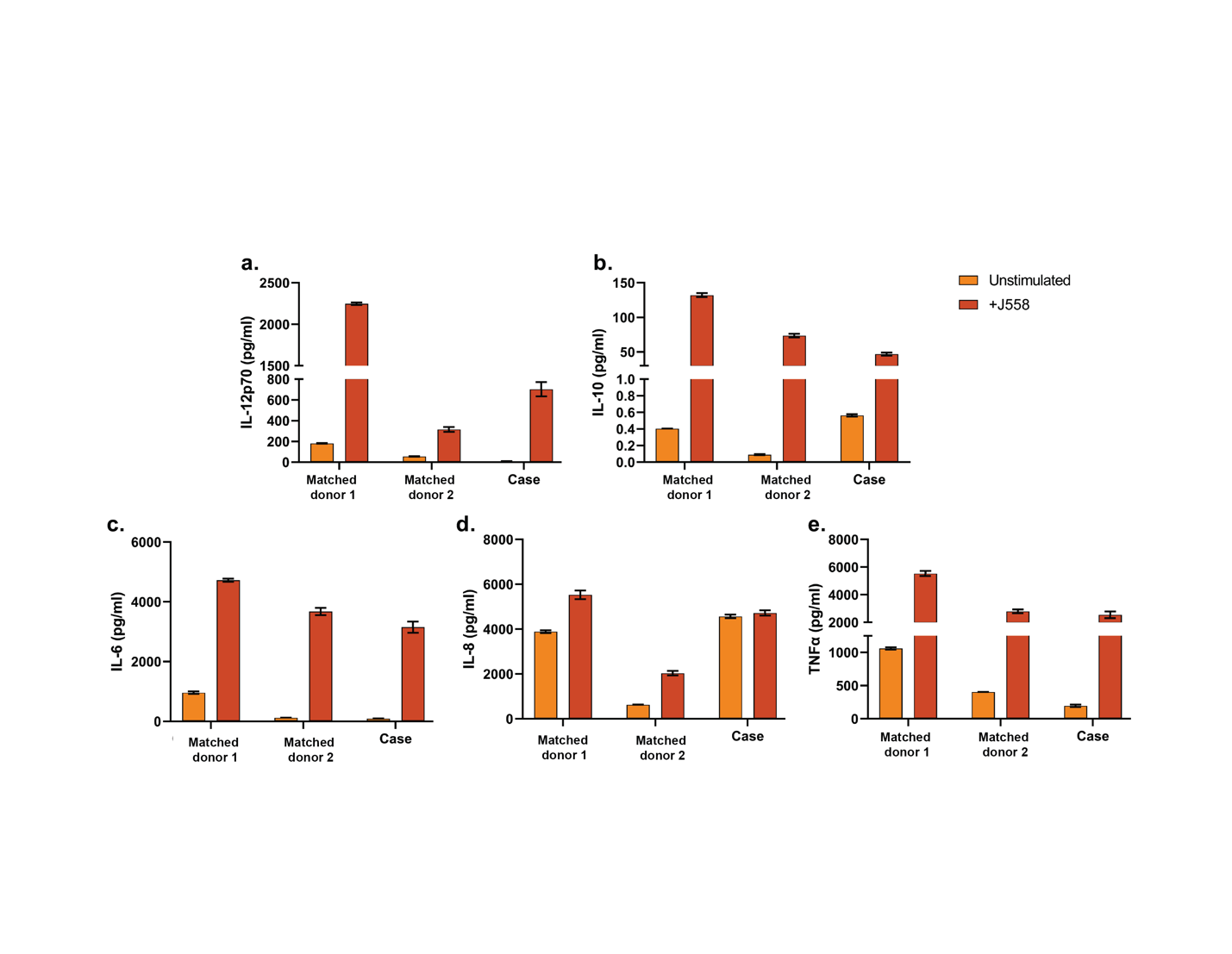


Fig. S10. Production of (a) IL-12p70, (b) IL-10, (c) IL-6, (d) IL-8, and (e) TNF-α by monocyte-derived DCs from the case and two matched donors, either unstimulated, or stimulated with J558, a CD40L-transfected cell line, for 24 hours. Cytokines in the supernatants of the cell culture were measured by multiplex electrochemiluminescence immunoassay.


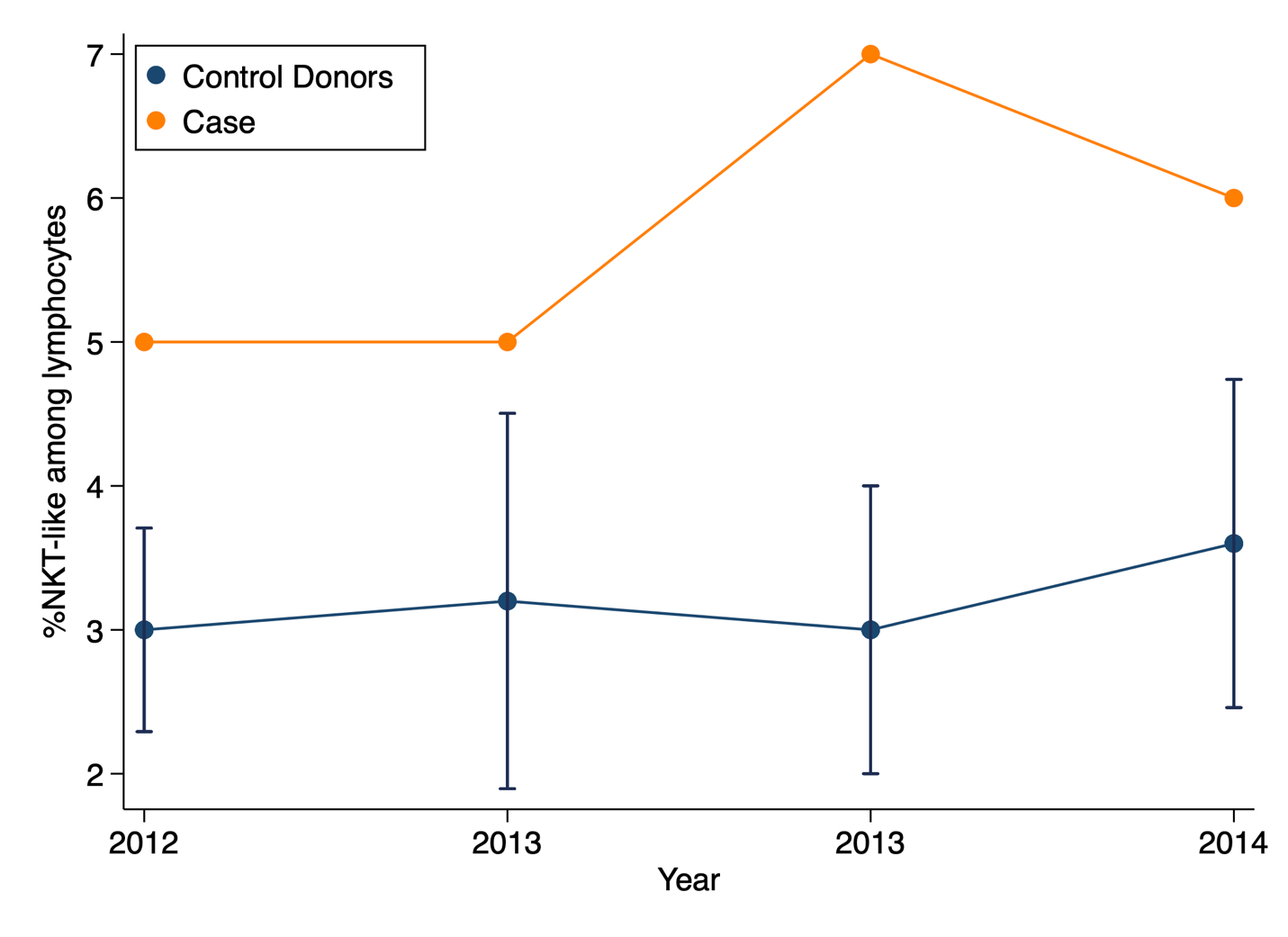


Fig. S11. Percentages of NKT-like (CD56+/CD16+CD3+) cells among lymphocytes of the case and of 5 matched control donors across two years. The orange circles represent data from the case; blue circles and error bars represent the means and standard deviations of the data from the control donors.

Supplementary Table 1. Antibodies used for immunophenotyping of NK cells

| Marker | Clone | Fluorochrome | Manufacturer | Specificity | Panels for experiment 3^a^ |
| --- | --- | --- | --- | --- | --- |
| live/dead | NA | Zombie Yellow | Biolegend | Exclude dead cells | 1 and 2 |
| CD3 | UCHT1 | Alexa Fluor 700 | BD Biosciences | Lineage marker for T Cells, exclude T cells | 1 and 2 |
| CD4 | RPA-T4 | Alexa Fluor 700 | BD Biosciences | Lineage marker for T cells and monocytes, exclude T cells | 1 and 2 |
| CD14 | M5E2 | Alexa Fluor 700 | BD Biosciences | Lineage marker for monocytes, exclude monocytes | 1 and 2 |
| CD19 | HIB19 | Alexa Fluor 700 | BD Biosciences | Lineage marker for B cells, exclude B cells | 1 and 2 |
| CD16 | 3G8 | BUV395 | BD Biosciences | NK cell phenotyping | 1 and 2 |
| CD56 | NCAM16.2 | BUV615 | BD Biosciences | NK cell phenotyping, identify CD56^bright^ and CD56^dim^ subsets | 1 and 2 |
| NKG2A | S19004C | APC Fire™750 | Biolegend | Inhibitory receptor, identify early-differentiated NK cells | 1 |
| NKG2C^b^ | S19005E | BV711/PE | Biolegend | Activating Receptor, identify memory-like or adaptive NK cells | 1 |
| NKG2D | 1D11 | BV480 | BD Biosciences | Activating Receptor, identify early-differentiated NK cells | 1 |
| CD2 | RPA-2.10 | BUV805 | BD Biosciences | High expression on adaptive and immature CD56^bright^ NK cells | 1 |
| CD94 | HP-3D9 | BV421 | BD Biosciences | Identify memory NK cells and early-differentiated NK cells | 1 and 2 |
| CD57 | HNK‐1 | APC | BD Biosciences | Identify adaptive NK cells | 1 |
| KIR2DL1 | HP-MA4 | PerCP Cy5.5 | BD Biosciences | Inhibitory receptor on NK cells | 1 |
| KIR2DL2 | CH-L | BUV496 | BD Biosciences | Inhibitory receptor on NK cells | 1 |
| KIR3DL1 | DX9 | BV650 | BD Biosciences | Inhibitory receptor on NK cells | 1 |
| Antibodies below require intracellular staining | | | | | |
| TBX-21 | 4B10 | PE-Cy7 | Biolegend | Transcription factor, identify total NK cells | 1 and 2 |
| FcRγ (FcɛRIγ) | poly | FITC | Sigma | Signaling adaptor associated with CD16, identify memory-like or adaptive NK cells | 1 |
| TCF-1^c^ | 7F11A10/C63D9 | PE | Biolegend/ Cell Signaling | Transcription factor, Identify memory NK cells | 2 |

All antibodies were included in one panel in experiments 1 and 2, but not experiment 3. ^a^ In experiment 3, immunophenotyping was split into two antibody panels because of dim staining of NKG2C-BV711; the antibodies used in each panel are indicated. ^b^ In experiments 1 and 2 NKG2C-BV711 was used, and in experiment 3 NKG2C-PE was used. ^c^ In experiments 1 and 2 TCF-1 clone 7F11A10 was used. We switched to clone C63D9 in experiment 3 because of inconsistent staining of clone 7F11A10.

Supplementary Table 2. Antibodies used for immunophenotyping of monocytes and DCs

| Marker | Fluorochrome | Clone | Manufacturer | Specificity | Sequential staining steps ^a^ |
| --- | --- | --- | --- | --- | --- |
| live/dead | BV575/Yellow | N/A | Thermo Fisher | Exclude dead cells | N/A |
| CX3CR1 | BB515 | 2A9-1 | BD | Chemokine receptors that are differentially expressed on monocyte subsets | Step 1, 30 minutes, 37℃ |
| CCR3 | RY586 | 5E8 | BD | Chemokine receptors that are differentially expressed on monocyte subsets | Step 1, 30 minutes, 37℃ |
| CXCR2 | PE-Dazzle 594 /APC-Fire750^b^ | 5E8/CXCR2 | Biolegend | Chemokine receptors that are differentially expressed on monocyte subsets | Step 2, 5 minutes, 37℃ |
| CD14 | BV650 | 63D3.rMAb | BD | Identify monocytes and CD14+CD163+ DC3 | Step 3, 5 minutes, 37℃ |
| CCR2 | BV421 | 48607 | BD | Chemokine receptors that are differentially expressed on monocyte subsets | Step 4, 5 minutes, 37℃ |
| CD3 | BUV805 | SK7 | BD | Lineage marker for T cells, exclude T cells | Step 5, 30 minutes, room temp |
| CD56 | BUV615 | NCAM16.2 | BD | Lineage marker for NK cells, exclude NK cells | Step 5, 30 minutes, room temp |
| CD19 | PE-Cy5 | HIB19 | BD | Lineage marker for B cells, exclude B cells | Step 5, 30 minutes, room temp |
| HLA-DR | RB613 | G46-6 | BD | Identify monocytes and DCs | Step 5, 30 minutes, room temp |
| CD88 | PE-Cy7 | S5/1 | Biolegend | Distinguish monocytes from DCs | Step 5, 30 minutes, room temp |
| CD89 | BUV661 | A59 | BD | Distinguish monocytes from DCs | Step 5, 30 minutes, room temp |
| CD16 | BUV496 | 3G8 | BD | Identify non-classical and intermediate monocytes | Step 5, 30 minutes, room temp |
| SLAN | APC | DD-1 | Miltenyi | Expressed on non-classical monocytes | Step 5, 30 minutes, room temp |
| CD123 | RB705 | 7G3 | BD | Expressed on pDCs and non-classical monocytes | Step 5, 30 minutes, room temp |
| CD1c | BUV395 | F10/21A3 | BD | Identify type 2 cDCs | Step 5, 30 minutes, room temp |
| CD303 | BV750 | V24-785 | BD | Identify pDCs | Step 5, 30 minutes, room temp |
| CD141 | BV786 | 1A4 | BD | Identify type 1 cDCs | Step 5, 30 minutes, room temp |
| CD11c | R718 | B-ly6 | BD | Identify classical DCs (cDCs) | Step 5, 30 minutes, room temp |
| CD163 | BUV737 | GHI/61 | BD | Scavenger receptor that is differentially expressed on monocyte subsets and on DC3 | Step 5, 30 minutes, room temp |

^a^ All antibodies were included in one panel. Sequential staining with antibodies against chemokine receptors and CD14 was performed according to Hally, *et. al.*[7]

^b^ CXCR2-PE-Dazzle 594 was used in experiment 1 and CXCR2-APC-Fire750 was used in experiment 2.

Supplementary Table 3. Percentages and absolute counts of NK cells and B cells among lymphocytes of the case, by study visit.

| **Year of Study Visit^a^** | **Percentage of NK Cells** | **Absolute count of NK Cells (cells/mm^3^)** | **Percentage of B Cells** | **Absolute count of B cells (cells/mm^3^)** |
| --- | --- | --- | --- | --- |
| **2012** | **3** | **82** | **16** | **437** |
| **2013** | **4** | **103** | **14** | **360** |
| **2013** | **5** | **135** | **12** | **324** |
| **2014** | **3** | **81** | **18** | **488** |
| **2017** | **4** | **128** |  |  |

^a^ Percentages of NK cells were measured routinely on fresh whole blood during study visits until 2014. Data from 2017 were obtained from cryopreserved PBMC.

Supplementary Table 4. Summary of expression of chemokine receptors and markers (percentages of positive cells) in monocyte subsets of the case and matched control donors.

| A | | Case | Controls | | | | | |  |  | |  |  |  | | |  |  |  |  |
| --- | --- | --- | --- | --- | --- | --- | --- | --- | --- | --- | --- | --- | --- | --- | --- | --- | --- | --- | --- | --- |
| Monocyte (%)^a^ | | 9.8 | 6.9 (0.8)^b^ | | | | | |  |  | |  |  |  | | |  |  |  |  |
| Monocyte (cells/uL) | | 706 | 573 (482) | | | | | |  |  | |  |  |  | | |  |  |  |  |
| Percentages among monocytes (%) | |  |  | | | | | |  |  | |  |  |  | | |  |  |  |  |
| Classical (CD14^+^CD89^+^) | | 91.9 (7.3) | 88.2 (3.2) | | | | | |  |  | |  |  |  | | |  |  |  |  |
| Intermediate (CD14^dim^ CD89^dim^) | | 4.1 (2.1) | 6.2 (3.3) | | | | | |  |  | |  |  |  | | |  |  |  |  |
| Nonclassical (CD14^-^CD89^-^) | | 3.4 (4.5) | 4.9 (1.8) | | | | | |  |  | |  |  |  | | |  |  |  |  |
| Staining of monocytes for markers: | | | | | | | | | | | | | | | | | | | | |
| B | Reported to be preferentially expressed on classical monocytes | | | | | | | | | | | | | | | | | | | |
|  | CCR2 | | | CCR3 | | | CXCR2 | | | | | | | | CD163 | | | | | |
|  | Case | Controls | | | Case | Controls | | Case | | | Controls | | | | | Case | | | | Controls |
| Classical | 84.5 (6.5) | 83.1 (13.7) | | | 14.1 (7.4) | 35.9 (20.7) | | 2.3 (1.6) | | | 6.1 (6.8) | | | | | 52.2 (7.6) | | | | 74.2 (35.1) |
| Intermediate | 27.7 (12.1) | 18.7 (10.8) | | | 7.5 (3.7) | 7.0 (7.1) | | 1.2 (1.2) | | | 1.9 (6.1) | | | | | 14.3 (4.0) | | | | 24.3 (14.4) |
| Nonclassical | 1.0 (1.0) | 0.8 (0.7) | | | 6.4 (2.3) | 7.4 (14.1) | | 1.1 (1.6) | | | 0.6 (1.1) | | | | | 0.9 (0.6) | | | | 2.7 (4.7) |
|  |  |  | | |  |  | |  | | |  | | | | |  | | | |  |
| C | Reported to be preferentially expressed on nonclassical monocytes | | | | | | | | | | | | | | | | | | |  |
|  | slan | | | CX3CR1 | | | CD123 | | | | | | | |  | | | |  | |
|  | Case | Controls | | | Case | Controls | | Case | | | Controls | | | | |  | | | |  |
| Classical | **0.1 (0.0)**^c^ | **0.0 (0.0)** | | | 0.7 (1.5) | 1.5 (10.4) | | 9.7 (0.1) | | | 11.3 (2.7) | | | | |  | | | |  |
| Intermediate | **36.5 (3.1)** | **9.5 (7.6)** | | | 54.5 (19.6) | 66.9 (40.7) | | 58.9 (8.6) | | | 58.4 (52.9) | | | | |  | | | |  |
| Nonclassical | **76.5 (0.8)** | **31.1 (18.8)** | | | 90.3 (2.1) | 82.7 (38.5) | | 78.2 (0.5) | | | 66.2 (90.7) | | | | |  | | | |  |
|  |  |  | | |  |  | |  | | |  | | | | |  | | | |  |
| D | Case | Control | | |  |  | |  | | |  | | | | |  | | | |  |
| Slan^+^ intermediate monocytes (cells/uL) | 10.4 (4.6) | 3.5 (4.3) | | |  |  | |  | | |  | | | | |  | | | |  |
| Slan^+^ nonclassical monocytes (cells/uL) | 14.7 (16.9) | 9.3 (10.0) | | |  |  | |  | | |  | | | | |  | | | |  |

^a^ Percentages and numbers of monocytes were measured at Quest Diagnostics ^b^ Median (interquartile range) of data compiled from two experiments. ^c^ Bold values indicate that the difference between the case and control donors was significant (p<.05).

Supplementary Table 5. Summary of serum concentration of sCD14 and sCD163 in the case and virally suppressed HIV+ men in the MACS

|  | Case | Other virally suppressed HIV+ men in the MACS | | Percentile of the case's values relative to the other virally suppressed MACS men |
| --- | --- | --- | --- | --- |
| Markers |  | N | Median (IQR) |  |
| sCD14 (ng/mL) | 3620.1^a^ | 1131 | 2553.2 (917.3) | 88 |
| sCD163 (ng/mL) | 440.44 | 598 | 660.8(341.1) | 16 |

^a^ sCD14 for the case and the other virally suppressed HIV+ men in the MACS was measured at the first visit where they were HIV virally suppressed.

Supplementary Table 6. Summary of percentages of peripheral DC subsets in the case and matched controls

| DC subsets | Case | Controls |
| --- | --- | --- |
| Total DC (%) | 9.1 | 11.4 (2.4)^a^ |
| CD123^+^CD11c^-^ CD303^+^(pDC) among DC(%) | 12.7 | 6 (5.0) |
| CCR2^–^ among pDC (%) | 21.7 | 51.7 (35.9) |
| CCR2^+^ among pDC (%) | 78.1 | 44.6 (33.5) |
| CD123^-^CD11c^+^ (cDC) among DC (%) | 85.9 | 91.8 (4.5) |
| CD141^+^CD1c- (cDC1) among cDC (%) | 3.2 | 2.8 (1.5) |
| CD141^-^CD1c^+^(cDC2) among cDC (%) | 62.8 | 60.7 (22.9) |
| CD1c^-^CD141^-^ among cDC (%) | 33.4 | 36.1 (24.2) |
| CD5^+^ among cDC2 (%) | 16.8 | 15.8 (10.1) |
| CD5^-^ among cDC2 (%) | 81 | 80.9 (10.6) |
| CD163^-^ CD14 ^–^ among CD5- cDC2 (%) | 20.7 | 19.8 (2.3) |
| CD163^+^ CD14^–^ among CD5- cDC2 (%) | 50.2 | 28.7 (12.3) |
| CD163^+^ CD14^+^ among CD5- cDC2 (%) | 25.3 | 45.9 (14.8) |
| CD163^-^ CD14^+^ among CD5- cDC2 (%) | 3.9 | 5.3 (5.9) |
| CD5^-^CD163^+^CD14^-^DC3 among cDC2 (%) | 25.5 | 14.1 (9.5) |
| CD5^-^CD163^+^CD14^+^ among cDC2 (%) | 12.9 | 1. (7.6) |

^a^ Median (IQR) of values obtained from three donors in experiment 2. Data from experiment 1 is not shown because of the low numbers of total DCs acquired.

Reference

1. Mair F, Liechti T. Comprehensive Phenotyping of Human Dendritic Cells and Monocytes. Cytometry A. 2021;99:231–42.

2. Sandblad KG, Jones P, Kostalla MJ, Linton L, Glise H, Winqvist O. Chemokine receptor expression on monocytes from healthy individuals. Clin Immunol. 2015;161:348–53.

3. Cros J, Cagnard N, Woollard K, Patey N, Zhang S-Y, Senechal B, et al. Human CD14dim Monocytes Patrol and Sense Nucleic Acids and Viruses via TLR7 and TLR8 Receptors. Immunity. 2010;33:375.

4. Ancuta P. A slan-based nomenclature for monocytes? Blood. 2015;126:2536–8.

5. Hamlin RE, Pienkos SM, Chan L, Stabile MA, Pinedo K, Rao M, et al. Sex differences and immune correlates of Long Covid development, symptom persistence, and resolution. Sci Transl Med. 2024;16:eadr1032.

6. Wong KL, Tai JJ-Y, Wong W-C, Han H, Sem X, Yeap W-H, et al. Gene expression profiling reveals the defining features of the classical, intermediate, and nonclassical human monocyte subsets. Blood. 2011;118:e16–31.

7. Hally KE, Ferrer-Font L, Pilkington KR, Larsen PD. OMIP 083: A 21-marker 18-color flow cytometry panel for in-depth phenotyping of human peripheral monocytes. Cytometry A. 2022;101:374–9.
